# Ultra-processed food consumption, cancer risk and cancer mortality: a large-scale prospective analysis within the UK Biobank

**DOI:** 10.1016/j.eclinm.2023.101840

**Published:** 2023-01-31

**Authors:** Kiara Chang, Marc J. Gunter, Fernanda Rauber, Renata B. Levy, Inge Huybrechts, Nathalie Kliemann, Christopher Millett, Eszter P. Vamos

**Affiliations:** a Public Health Policy Evaluation Unit, Imperial College London, London, W6 8RP, United Kingdom; b Nutrition and Metabolism Branch, International Agency for Research on Cancer, 69372, Lyon, France; c Center for Epidemiological Research in Nutrition and Health, School of Public Health, University of São Paulo, São Paulo, 01246-904, Brazil; d Department of Preventive Medicine, School of Medicine, University of São Paulo, São Paulo, 01246-904, Brazil; e NOVA National School of Public Health, Public Health Research Centre, Comprehensive Health Research Center, CHRC, NOVA University Lisbon, Lisbon, Portugal

**Keywords:** Ultra-processed food, Cancer incidence, Cancer mortality, Prospective cohort, United Kingdom

## Abstract

**Background:**

Global dietary patterns are increasingly dominated by relatively cheap, highly palatable, and ready-to-eat ultra-processed foods (UPFs). However, prospective evidence is limited on cancer development and mortality in relation to UPF consumption. This study examines associations between UPF consumption and risk of cancer and associated mortality for 34 site-specific cancers in a large cohort of British adults.

**Methods:**

This study included a prospective cohort of UK Biobank participants (aged 40–69 years) who completed 24-h dietary recalls between 2009 and 2012 (N = 197426, 54.6% women) and were followed up until Jan 31, 2021. Food items consumed were categorised according to their degree of food processing using the NOVA food classification system. Individuals’ UPF consumption was expressed as a percentage of total food intake (g/day). Prospective associations were assessed using multivariable Cox proportional hazards models adjusted for baseline socio-demographic characteristics, smoking status, physical activity, body mass index, alcohol and total energy intake.

**Findings:**

The mean UPF consumption was 22.9% (SD 13.3%) in the total diet. During a median follow-up time of 9.8 years, 15,921 individuals developed cancer and 4009 cancer-related deaths occurred. Every 10 percentage points increment in UPF consumption was associated with an increased incidence of overall (hazard ratio, 1.02; 95% CI, 1.01–1.04) and specifically ovarian (1.19; 1.08–1.30) cancer. Furthermore, every 10 percentage points increment in UPF consumption was associated with an increased risk of overall (1.06; 1.03–1.09), ovarian (1.30; 1.13–1.50), and breast (1.16; 1.02–1.32) cancer-related mortality.

**Interpretation:**

Our UK-based cohort study suggests that higher UPF consumption may be linked to an increased burden and mortality for overall and certain site-specific cancers especially ovarian cancer in women.

**Funding:**

The 10.13039/501100000289Cancer Research UK and 10.13039/501100000321World Cancer Research Fund.

**Research in context**

**Evidence before this study**

We searched PubMed using combinations of search terms such as “ultra”, “industrial”, “processed”, “food”, “drink”, and “cancer” on 10 September 2022 with no date or language restrictions. Our search results showed limited prospective evidence for the association between ultra-processed consumption and cancer outcomes. To date, only incidence of the most common cancer sites including colorectal, breast, and prostate cancer has been examined and the cohort studies that assessed total cancer mortality may be potentially limited by small sample size. No previously published cohort study has assessed incidence and mortality for a comprehensive range of site-specific cancers in relation to ultra-processed food consumption, and there is currently no data from the UK despite it is one of the world's leading consumers of ultra-processed foods.

**Added value of this study**

Our study provides the first most comprehensive assessment for the prospective associations between ultra-processed food consumption and risk of overall and 34 site-specific cancer incidence and associated mortality. Our findings show that higher consumption of ultra-processed foods is associated with a greater risk of overall cancer and specifically ovarian and brain cancer, as well as increased risk of overall, ovarian, and breast cancer-associated mortality. These associations persisted after adjustment for a range of socio-demographic, smoking status, physical activity, and key dietary factors.

**Implications of all the available evidence**

Cancer has surpassed cardiovascular disease as the leading cause of premature death in many high-income countries while cancer burden is rising most rapidly in low and middle-income countries. Our study adds important prospective evidence linking ultra-processed food to an increased risk of adverse cancer outcomes. Lowering consumption of ultra-processed foods in dietary patterns may be beneficial for the prevention and risk reduction of overall and certain site-specific cancers.

## Introduction

The global burden of cancer continues to rise, with incident cases projected to increase from 19.3 to 28.4 million by 2040.^1^ Cancer is responsible for one in six deaths globally and has surpassed cardiovascular disease as the leading cause of premature mortality in many high-income countries.^2^^,^^3^ However, at least 50% of cancer cases could be potentially preventable and an unhealthy diet is a key modifiable risk factor.^2^^,^^4^ There are growing concerns over the potential harmful health effects of ultra-processed foods (UPF) - foods that are industrial formulations made by assembling industrially-derived food substances and food additives through a sequence of extensive industrial processes.^5^ UPFs contain little or no whole foods and are often energy dense, high in salt, sugar and fat, low in fibre, and liable to overconsumption.^5^ They are aggressively marketed with strong brands to promote consumption and are gradually displacing traditional dietary patterns based on fresh and minimally processed foods.^5^

The global consumption of UPFs has been rising rapidly in recent decades, and the UK and US are leading consumers with UPFs exceeding 50% of daily calorie intake.^6^^,^^7^ Evidence has been accumulating on the associations of higher UPF consumption and increased risks of adverse health outcomes including obesity, type 2 diabetes (T2D), and all-cause mortality.^7^ However, prospective evidence on the association of UPF consumption and cancer outcomes is limited to a few studies that assessed the incidence of common cancers or total cancer mortality.^8^, ^9^, ^10^, ^11^, ^12^ Besides their poorer nutritional composition, UPFs may additionally increase cancer risk through neo-formed contaminants during industrial processing, use of some controversial food additives, and certain materials of packaging implicated in exhibiting carcinogenic and/or endocrine-disrupting properties.^13^, ^14^, ^15^ Therefore, this study aims to provide a comprehensive assessment of the association between UPF consumption and risk of overall and 34 site-specific cancer incidence and mortality in a large and contemporary cohort of British adults, in a country with prominently high UPF consumption.

## Methods

### Data source

The UK Biobank is a large prospective study with over half a million participants aged 40–69 years recruited across England, Scotland, and Wales between 2007–2010.^16^ During recruitment, participants completed questionnaires regarding socio-demographic, lifestyle and psychosocial characteristics, and had objective anthropometrics measurements, medical history and medication use recorded/verified by trained research staff.^16^ Dietary intakes were assessed using a web-based, self-administered 24-h recall which was conducted five times between 2009 and 2012. This 24-h recall has been validated against an interviewer-administered 24-h recall showing similar recordings of food items as well as estimated energy and nutrient intakes.^17^ The UK Biobank received ethical approval from the North West Multi-centre Research Ethics Committee (21/NW/0157) and data access was granted by the UK Biobank's Access Sub-committee.^16^ All participants provided written informed consent, allowing for prospective data linkage to health records. The ethical review boards from the International Agency for Research on Cancer (IARC) approved the study.

### Dietary exposure and degree of industrial food processing

The derivation of individual dietary consumption by the degree of industrial food processing has been documented in detail elsewhere.^18^ In brief, we applied the NOVA food classification to 24-h recall data assigning each food and beverage item to one of the four main food groups according to their extent and purpose of food processing^5^: (1) unprocessed or minimally processed foods, e.g. fruit, vegetables, milk and meat; (2) processed culinary ingredients, e.g. sugar, vegetable oils and butter; (3) processed foods, e.g. canned vegetables in brine, freshly made breads and cheeses; and (4) UPFs, e.g. soft drinks, mass-produced industrial-processed breads, sweet or savoury packaged snacks, breakfast ‘cereals’, reconstituted meat products and ready-to-eat/heat foods.

The main exposure variable was individuals’ UPF consumption, expressed as a percentage of UPF content in the total diet (g/day), averaged across multiple 24-h recalls. This weight ratio was preferred over an energy ratio as it better captures UPFs with zero or low-calorie content such as artificially sweetened beverages. We further categorised individuals into UPF consumption quartiles. In sensitivity analysis, we computed UPF consumption as a percentage of total energy intake (kcal/day) for comparison.

### Outcome measures

Incident cancer cases were identified through data linkage to national cancer and mortality registries, provided by the National Health Service (NHS) Digital for participants in England and Wales, and NHS Central Register for participants in Scotland.^16^ Cancer registries were coded in the 10th revision of the International Classification of Diseases (ICD-10) and the third edition of ICD for Oncology (ICD-O-3) morphology codes where appropriate, and were available up to 31 July 2019 for England and Wales, and 31 October 2015 for Scotland. Mortality registries were coded using ICD-10 and available up to 31 January 2021. Cancer deaths were defined as primary/underlying cause of death. We examined all cancers (C00–C97, except for non-melanoma skin cancer C44) and 34 site-specific cancers. Detailed list, coding and case numbers for each site-specific cancer are presented in Appendix Table S1 for cancer incidence and Appendix Table S2 for cancer mortality. Cancers with small case numbers (n < 40, e.g. hypopharynx, larynx) are presented but excluded from subsequent analyses.

### Study covariates

Covariates included age, sex (male, female), ethnicity (white, mixed, south Asian, Black), height (cm), family history of cancer (yes, no), smoking status (never smoked, ex-smoker, current smoker), physical activity level (low, moderate, high), average household income (<£18,000, £18,000-£30,999, £31,000-£51,999, >£52,000), highest educational attainment (university degree, A levels or equivalent, O levels or equivalent, vocational qualification, none of the above), Index of Multiple Deprivation (IMD) quintile, geographical region, alcohol intake (g/day), body mass index (BMI) categorised as underweight (<18.5 kg/m^2^), normal (18.5–24.9 kg/m^2^), overweight (25–29.9 kg/m^2^), or obese (≥30 kg/m^2^), total energy intake (kcal/day), and female-specific characteristics including menopausal status (pre-menopausal, post-menopausal, unsure because of hysterectomy, unsure because of other reason, unknown), use of oral contraceptives (never, ever, unknown), use of hormone-replacement therapy (never, ever, unknown), and parity (0, 1–2, ≥3, unknown). IMD is a composite measure of deprivation for each small area of the UK based on participants' postcode, and we derived IMD quintiles based on deprivation scores.^16^ Additional covariates considered in sensitivity analysis included intake of sodium, total fat, carbohydrate, red meat, processed meat, fibre, and calcium; and presence of diabetes, cardiovascular disease (angina, myocardial infarction, and stroke), depression, and hypertension at baseline. Missing data were under 3% except for physical activity (15.1% missing) and average household income (9.4% missing). We used multiple imputation by chained equation with 10 imputed datasets to estimate missing covariate data under assumption of missing at random and the analytical results were combined using Rubin's rule.

### Statistical analysis

The study cohort included 197,426 UK Biobank participants with 24-h recall data (78,330 [39.7%], 45,137 [22.9%], 39,841 [20.2%], 28,654 [14.5%], and 5464 [2.8%] with one to five recalls, respectively) after excluding 12,680 individuals with pre-existing cancers, 173 individuals who were pregnant/unsure, and 668 individuals with an implausible total daily energy intake outside of 500–5000 kcal/day (Appendix Fig. S1). We compared participants’ baseline characteristics by quartile of UPF consumption using analysis of variance for continuous and χ2 test for categorical variables. Furthermore, we estimated and graphically presented the mean percentage of daily food intake (or daily energy intake) for each NOVA subgroup.

We used Cox proportional hazards regression with age as the underlying time metric to estimate the hazard ratios (HRs) and 95% confidence intervals (CIs) for the association between UPF consumption and each cancer outcome. Participants contributed person time until the date of cancer incidence/mortality, death of non-cancer causes, lost to follow-up, or end of study period, whichever occurred first. We built four models in incremental steps: Model 1 included age (timescale) and UPF consumption, stratified by sex; Model 2 additionally included ethnicity, smoking status, physical activity, average household income, highest educational attainment, alcohol intake, and additionally stratified by height, family history of cancer, IMD, and geographical region; Model 3 additionally included BMI category; Model 4 (final model) additionally included daily energy intake. For breast, uterus, and ovarian cancer outcomes, we additionally included in Model 2: baseline menopausal status, use of oral contraceptives, use of hormone replacement therapy, and parity. We performed separate Cox regression for each measurement of UPF consumption: continuous per 10% absolute increment in UPF content, categorical UPF quartiles, and trend using ordinal UPF quartiles. The proportional hazards assumption was evaluated by tests based on Schoenfeld residuals. Potential non-linearity in the association were examined by considering higher order polynomials of UPF consumption and restricted cubic spline function but none were identified. Lung cancer outcomes were additionally stratified by smoking status, and head and neck cancer incidence was stratified by smoking status and alcohol consumption in an exploratory analysis.

The following sensitivity analyses were performed based on Model 4 (final model): (i) additionally adjusting for key dietary factors including sodium, total fat, and carbohydrate intake while total energy intake was removed due to high correlation (>0.74) between total energy intake and these nutrients. Colorectal cancer analyses were additionally adjusted for red meat, processed meat, fibre, and calcium intake; (ii) additionally adjusting for intake of sodium, trans fat, and free sugars while total energy intake was removed from the model (results were consistent when intake of saturated fat was adjusted for instead of trans fat but they were not simultaneously included in the model due to a high correlation of 0.79); (iii) additionally adjusting for fruit and vegetable intake; (iv) removing (ultra-processed) alcohol intake from the UPF exposure and also deducting the energy contribution of alcohol consumption from the total energy intake; (v) additionally adjusting for baseline presence of diabetes, CVD, depression, and hypertension; (vi) additionally adjusting for number of 24-h recalls; (vii) excluding participants with follow-up time <2 years.

All statistical analyses were performed using Stata SE version 12.1. All tests were two-sided and a *P* < 0.05 was considered statistically significant. A more conservative Bonferroni-corrected *P* < 0.05 accounting for multiple comparisons (computed by multiplying the original *P*-value by 45 for analysis of cancer incidence and 28 for analysis of cancer mortality) is additionally presented.

### Role of the funding source

The funders had no role in study design, data collection, data analysis, interpretation of study findings, writing of the report, or decision to submit the paper for publication. All authors had full access to all the data in the study, approved the final manuscript, and accept responsibility for the decision to submit for publication.

## Results

**Fig. 1:**
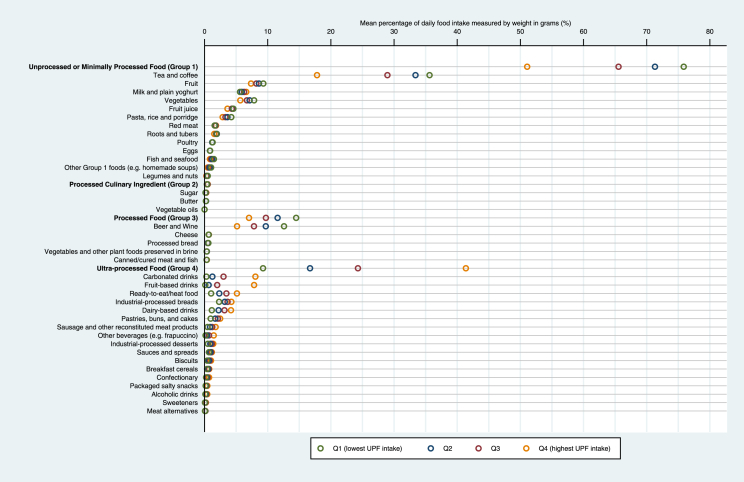
**Sources of NOVA subgroups in the total diet by quartile of ultra-processed food consumption**. UPF, ultra-processed food. UPF consumption was defined as the percentage of its weight contribution relative to total food intake measured in g/day. Study participants were further categorised into quartiles (Q1-Q4 represents lowest to highest quartile of UPF consumption).

The mean age of study participants was 58.0 (SD, 8.0) years and 54.6% (107,919/197,426) were female (Table 1). The mean UPF consumption was 22.9% (SD, 13.3%) in the total diet (g/day) and ranged from 9.2% (3.0%) to 41.4% (11.1%) among participants with the lowest to highest UPF consumption (quartile 1–4) (Fig. 1). Participants with highest compared with lowest UPF consumption quartile were younger and less likely to have a family history of cancer. Proportions of participants with BMI levels indicating obesity, lower physical activity, lower socio-economic status (lower household income, educational attainment, and living in most deprived neighbourhoods), and key nutritional factors (total energy, sodium, carbohydrate, and total fat intake) steadily increased along UPF consumption groups from the lowest to highest quartile.

**Table 1:** Baseline characteristics by quartile of ultra-processed food consumption among UK Biobank participants.

		Quartile of ultra-processed food consumption^a^				
	Overall	1 (lowest)	2	3	4 (highest)	*P* value^b^
	197,426	49,357	49,356	49,357	49,356	
**Proportion of UPF in total diet, %g/day**						
Mean (SD)	22.9 (13.3)	9.2 (3.0)	16.7 (1.9)	24.3 (2.6)	41.4 (11.1)	<0.001
Range	0%–100%	0%–13.4%	13.5%–20.0%	20.1%–29.4%	29.5%–100%	
**Age, year**						
Mean (SD)	58.0 (8.0)	58.8 (7.5)	58.7 (7.8)	58.1 (8.0)	56.4 (8.3)	<0.001
**Sex, n (%)**						
Male	89,507 (45.3%)	21,085 (42.7%)	21,998 (44.5%)	22,980 (46.5%)	23,444 (47.4%)	<0.001
Female	107,919 (54.6%)	28,272 (57.2%)	27,358 (55.4%)	26,377 (53.4%)	25,912 (52.5%)	
**Ethnicity, n (%)**						
White	188,189 (95.3%)	47,305 (95.8%)	47,543 (96.3%)	47,154 (95.5%)	46,187 (93.5%)	<0.001
Mixed	1182 (0.5%)	270 (0.5%)	269 (0.5%)	306 (0.6%)	337 (0.6%)	
South Asian	2836 (1.4%)	559 (1.1%)	534 (1.0%)	708 (1.4%)	1035 (2.0%)	
Black	2469 (1.2%)	476 (0.9%)	397 (0.8%)	524 (1.0%)	1072 (2.1%)	
Chinese or other	2026 (1.0%)	565 (1.1%)	439 (0.8%)	483 (0.9%)	539 (1.0%)	
Missing	724 (0.3%)	182 (0.3%)	174 (0.3%)	182 (0.3%)	186 (0.3%)	
**Family history of cancer, n (%)**						
No	127,153 (64.4%)	31,477 (63.7%)	31,392 (63.6%)	31,697 (64.2%)	32,587 (66.0%)	<0.001
Yes	70,273 (35.5%)	17,880 (36.2%)	17,964 (36.3%)	17,660 (35.7%)	16,769 (33.9%)	
**Height, cm**						
Mean (SD)	169.2 (9.1)	168.9 (9.0)	169.2 (9.1)	169.4 (9.2)	169.3 (9.2)	<0.001
Missing (n, %)	369 (0.1%)	95 (0.1%)	82 (0.1%)	82 (0.1%)	110 (0.2%)	
**Body mass index, n (%)**						
Underweight (<18.5 kg/m^2^)	1071 (0.5%)	325 (0.6%)	274 (0.5%)	278 (0.5%)	194 (0.3%)	<0.001
Normal (18.5–24.9 kg/m^2^)	72,550 (36.7%)	20,930 (42.4%)	19,890 (40.2%)	17,698 (35.8%)	14,032 (28.4%)	
Overweight (25–29.9 kg/m^2^)	81,821 (41.4%)	20,142 (40.8%)	20,477 (41.4%)	20,878 (42.2%)	20,324 (41.1%)	
Obese (≥30 kg/m^2^)	41,510 (21.0%)	7832 (15.8%)	8597 (17.4%)	10,411 (21.0%)	14,670 (29.7%)	
Missing	474 (0.2%)	128 (0.2%)	118 (0.2%)	92 (0.1%)	136 (0.2%)	
**Smoking status, n (%)**						
Never smoked	111,814 (56.6%)	26,724 (54.1%)	28,137 (57.0%)	28,466 (57.6%)	28,487 (57.7%)	<0.001
Ex-smoker	69,545 (35.2%)	18,558 (37.5%)	17,647 (35.7%)	17,153 (34.7%)	16,187 (32.7%)	
Current smoker	15,622 (7.9%)	3966 (8.0%)	3475 (7.0%)	3640 (7.3%)	4541 (9.2%)	
Missing	445 (0.2%)	109 (0.2%)	97 (0.1%)	98 (0.1%)	141 (0.2%)	
**Physical activity level, n (%)**						
High	66,010 (33.4%)	17,775 (36.0%)	16,550 (33.5%)	16,243 (32.9%)	15,442 (31.2%)	<0.001
Moderate	70,730 (35.8%)	17,989 (36.4%)	18,247 (36.9%)	17,832 (36.1%)	16,662 (33.7%)	
Low	30,712 (15.5%)	6680 (13.5%)	7271 (14.7%)	7706 (15.6%)	9055 (18.3%)	
Missing	29,974 (15.1%)	6913 (14.0%)	7288 (14.7%)	7576 (15.3%)	8197 (16.6%)	
**Average household income, n (%)**						
≥£52,000	57,007 (28.8%)	16,866 (34.1%)	14,789 (29.9%)	13,443 (27.2%)	11,909 (24.1%)	<0.001
£31,000-£51,999	51,087 (25.8%)	12,461 (25.2%)	12,939 (26.2%)	12,809 (25.9%)	12,878 (26.0%)	
£18,000-£30,999	43,125 (21.8%)	9779 (19.8%)	10,771 (21.8%)	11,274 (22.8%)	11,301 (22.8%)	
<£18,000	27,453 (13.9%)	5801 (11.7%)	6400 (12.9%)	7099 (14.3%)	8153 (16.5%)	
Missing	18,754 (9.4%)	4450 (9.0%)	4457 (9.0%)	4732 (9.5%)	5115 (10.3%)	
**Highest educational attainment, n (%)**						
College/University degree	85,651 (43.3%)	25,215 (51.0%)	22,982 (46.5%)	20,523 (41.5%)	16,931 (34.3%)	<0.001
A/AS levels	26,219 (13.2%)	6249 (12.6%)	6507 (13.1%)	6678 (13.5%)	6785 (13.7%)	
O levels/GCSE/CSE	49,222 (24.9%)	10,055 (20.3%)	11,347 (22.9%)	12,699 (25.7%)	15,121 (30.6%)	
Vocational qualification	19,239 (9.7%)	4327 (8.7%)	4679 (9.4%)	4953 (10.0%)	5280 (10.6%)	
None of the above	16,194 (8.2%)	3286 (6.6%)	3668 (7.4%)	4286 (8.6%)	4954 (10.0%)	
Missing	901 (0.4%)	225 (0.4%)	173 (0.3%)	218 (0.4%)	285 (0.5%)	
**Index of Multiple Deprivation, n (%)**						
Quintile 1 (Least deprived)	42,582 (21.5%)	11,248 (22.7%)	11,512 (23.3%)	10,816 (21.9%)	9006 (18.2%)	<0.001
Quintile 2	42,606 (21.5%)	11,184 (22.6%)	11,059 (22.4%)	10,723 (21.7%)	9640 (19.5%)	
Quintile 3	40,232 (20.3%)	10,092 (20.4%)	10,229 (20.7%)	10,177 (20.6%)	9734 (19.7%)	
Quintile 4	37,352 (18.9%)	9008 (18.2%)	8894 (18.0%)	9346 (18.9%)	10,104 (20.4%)	
Quintile 5 (Most deprived)	29,679 (15.0%)	6549 (13.2%)	6435 (13.0%)	7118 (14.4%)	9577 (19.4%)	
Missing	4975 (2.5%)	1276 (2.5%)	1227 (2.4%)	1177 (2.3%)	1295 (2.6%)	
**Geographical region, n (%)**						
London	41,106 (20.8%)	12,218 (24.7%)	10,409 (21.0%)	9467 (19.1%)	9012 (18.2%)	<0.001
South East	16,139 (8.1%)	4371 (8.8%)	4169 (8.4%)	4021 (8.1%)	3578 (7.2%)	
South West	20,527 (10.3%)	5326 (10.7%)	5177 (10.4%)	5137 (10.4%)	4887 (9.9%)	
East Midlands	11,276 (5.7%)	2518 (5.1%)	2891 (5.8%)	2979 (6.0%)	2888 (5.8%)	
West Midlands	16,894 (8.5%)	3624 (7.3%)	3887 (7.8%)	4364 (8.8%)	5019 (10.1%)	
Yorkshire and the Humber	32,096 (16.2%)	7630 (15.4%)	8174 (16.5%)	8199 (16.6%)	8093 (16.3%)	
North East	18,735 (9.4%)	4011 (8.1%)	4542 (9.2%)	4780 (9.6%)	5402 (10.9%)	
North West	25,330 (12.8%)	6001 (12.1%)	6344 (12.8%)	6444 (13.0%)	6541 (13.2%)	
Wales	5715 (2.8%)	1323 (2.6%)	1413 (2.8%)	1467 (2.9%)	1512 (3.0%)	
Scotland	9608 (4.8%)	2335 (4.7%)	2350 (4.7%)	2499 (5.0%)	2424 (4.9%)	
**Presence of diabetes, n (%)**						
No	189,062 (95.7%)	47,730 (96.7%)	47,643 (96.5%)	47,360 (95.9%)	46,329 (93.8%)	<0.001
Yes	8364 (4.2%)	1627 (3.2%)	1713 (3.4%)	1997 (4.0%)	3027 (6.1%)	
**Presence of high blood pressure, n (%)**						
No	146,740 (74.3%)	37,523 (76.0%)	37,236 (75.4%)	36,562 (74.0%)	35,419 (71.7%)	<0.001
Yes	50,686 (25.6%)	11,834 (23.9%)	12,120 (24.5%)	12,795 (25.9%)	13,937 (28.2%)	
**Presence of cardiovascular disease, n (%)**						
No	188,757 (95.6%)	47,487 (96.2%)	47,290 (95.8%)	47,144 (95.5%)	46,836 (94.8%)	<0.001
Yes	8669 (4.3%)	1870 (3.7%)	2066 (4.1%)	2213 (4.4%)	2520 (5.1%)	
**Presence of depression, n (%)**						
No	188,016 (95.2%)	47,301 (95.8%)	47,172 (95.5%)	47,019 (95.2%)	46,524 (94.2%)	<0.001
Yes	9410 (4.7%)	2056 (4.1%)	2184 (4.4%)	2338 (4.7%)	2832 (5.7%)	
**Nutritional factors, mean (SD)**						
Total energy intake, kcal/day	2044.0 (614.0)	1817.5 (527.0)	2003.7 (544.5)	2127.7 (595.0)	2227.3 (697.3)	<0.001
Alcohol intake, g/day	17.6 (18.8)	23.1 (21.8)	18.8 (18.6)	16.0 (16.9)	12.5 (15.7)	<0.001
Carbohydrate intake, g/day	261.2 (90.3)	216.3 (69.9)	251.2 (74.4)	276.4 (85.0)	301.0 (105.2)	<0.001
Total fat intake, g/day	67.6 (27.1)	57.6 (23.6)	66.4 (24.3)	71.3 (26.4)	75.0 (30.2)	<0.001
Sodium intake, mg/day	1904.2 (769.4)	1511.4 (597.3)	1825.6 (639.6)	2027.4 (718.9)	2252.5 (887.9)	<0.001
Fibre intake, g/day	25.1 (10.7)	23.2 (9.9)	25.0 (10.0)	26.1 (10.7)	26.1 (11.8)	<0.001
Red meat intake, g/day	42.4 (56.9)	43.1 (58.7)	43.2 (55.2)	42.3 (54.9)	41.0 (58.7)	<0.001
Processed meat intake, g/day	35.1 (52.0)	22.3 (36.5)	32.0 (45.5)	39.0 (53.6)	47.0 (65.0)	<0.001
Fruit intake, g/day	216.7 (174.0)	255.0 (194.5)	227.9 (169.0)	208.3 (160.9)	175.7 (159.6)	<0.001
Vegetable intake, g/day	174.1 (149.9)	211.8 (173.0)	185.2 (144.9)	166.7 (137.8)	132.6 (129.0)	<0.001
**Whether had menopause**^c^**, n** (**%)**						
Pre-menopausal	29,140 (27.0%)	6622 (23.4%)	6783 (24.7%)	7213 (27.3%)	8522 (32.8%)	<0.001
Post-menopausal	62,536 (57.9%)	17,752 (62.7%)	16,625 (60.7%)	15,185 (57.5%)	12,974 (50.0%)	
Unsure because of hysterectomy	11,120 (10.3%)	2702 (9.5%)	2726 (9.9%)	2749 (10.4%)	2943 (11.3%)	
Unsure because of other reason	4976 (4.6%)	1158 (4.0%)	1191 (4.3%)	1199 (4.5%)	1428 (5.5%)	
Unknown	147 (0.13%)	38 (0.13%)	33 (0.12%)	31 (0.11%)	45 (0.17%)	
**Ever taken oral contraceptive pill**^c^**, n (%)**						
Never	16,051 (14.8%)	4042 (14.2%)	4205 (15.3%)	3978 (15.0%)	3826 (14.7%)	0.007
Ever	91,664 (84.9%)	24,178 (85.5%)	23,103 (84.4%)	22,357 (84.7%)	22,026 (85.0%)	
Unknown	204 (0.1%)	52 (0.1%)	50 (0.1%)	42 (0.1%)	60 (0.2%)	
**Ever used hormone-replacement therapy**^c^**, n (%)**						
Never	68,145 (63.1%)	17,500 (61.8%)	17,069 (62.3%)	16,674 (63.2%)	16,902 (65.2%)	<0.001
Ever	39,525 (36.6%)	10,704 (37.8%)	10,225 (37.3%)	9656 (36.6%)	8940 (34.5%)	
Unknown	249 (0.2%)	68 (0.2%)	64 (0.2%)	47 (0.1%)	70 (0.2%)	
**Parity**^c^**, n (%)**						
0	23,559 (21.8%)	6184 (21.8%)	5798 (21.1%)	5634 (21.3%)	5943 (22.9%)	<0.001
1–2	61,078 (56.5%)	15,760 (55.7%)	15,557 (56.8%)	15,132 (57.3%)	14,629 (56.4%)	
≥3	23,191 (21.4%)	6301 (22.2%)	5980 (21.8%)	5594 (21.2%)	5316 (20.5%)	
Unknown	91 (0.08%)	27 (0.09%)	23 (0.08%)	17 (0.06%)	24 (0.09%)	

UPF, ultra-processed food; SD, standard deviation.

a UPF consumption was defined as the percentage of its weight contribution relative to total food intake measured in g/day. Study participants were further categorized into quartiles (Q1-Q4 represents lowest to highest quartile of UPF consumption).

b χ2 tests (for categorical variables) and ANOVA tests (for continuous variables) were used to compare cohort characteristics across UPF consumption quartiles.

c Additional characteristics among female participants (n = 107919).

### UPF consumption and cancer incidence

A total of 15,921 incident cancer cases developed during 1,890,187 person-years of follow-up (median, 9.8 years; interquartile range [IQR], 9.4–10.6 years). The age-sex adjusted Cox regression models showed an elevated risk of cancer incidence with increasing levels of UPF consumption for multiple cancer sites (Appendix Fig. S2). In fully adjusted models (Table 2), statistically significant associations persisted for overall cancer (HR, 1.02; 95% CI, 1.01–1.04) and ovarian cancer in females (HR, 1.19; 1.08–1.30) per 10 percentage points increment in the UPF content of total diet. These findings remained consistent and robust to sensitivity analyses and Bonferroni correction (Table 2, Appendix Table S3). Hazard ratio for incident gastrointestinal cancer was borderline statistically significant but after further adjustment for comorbidities was no longer significant.

**Table 2:** Association between ultra-processed food consumption in the total diet and cancer incidence.

Cancer site	Number of incident cases	per 10% increment in UPF intake^a^ HR (95% CI)	Quartile of UPF consumption^a^				*P*_Trend_
			Q1 (lowest)	Q2	Q3	Q4 (highest)	
			Ref	HR (95% CI)	HR (95% CI)	HR (95% CI)	
**All cancers**	15,921	1.02 (1.01–1.04) d	1	0.98 (0.94–1.03)	0.99 (0.94–1.04)	1.07 (1.02–1.14)^d^	0.01
**Head and Neck**	342	0.89 (0.80–1.00)	1	0.71 (0.52–0.98)^c^	0.64 (0.45–0.89)^c^	0.59 (0.41–0.85)^d^	0.003
Oral cavity	106	0.91 (0.76–1.08)	1	0.93 (0.52–1.65)	0.65 (0.35–1.22)	0.58 (0.30–1.13)	0.07
Oropharynx	86	0.89 (0.71–1.11)	1	0.78 (0.41–1.50)	0.60 (0.30–1.20)	0.53 (0.25–1.13)	0.08
**Gastrointestinal**	2937	1.03 (1.00–1.07)^c^	1	0.94 (0.84–1.06)	0.99 (0.88–1.12)	1.07 (0.94–1.21)	0.20
**Oesophagus**	283	1.08 (0.97–1.21)	1	1.06 (0.74–1.53)	1.06 (0.73–1.56)	1.21 (0.80–1.81)	0.38
Adenocarcinoma	186	1.05 (0.91–1.20)	1	1.01 (0.64–1.59)	1.04 (0.65–1.67)	1.09 (0.65–1.81)	0.73
Squamous cell carcinoma	67	0.96 (0.74–1.24)	1	1.07 (0.53–2.18)	0.85 (0.39–1.84)	0.77 (0.31–1.87)	0.49
**Stomach**	189	1.08 (0.95–1.22)	1	0.92 (0.57–1.50)	0.95 (0.59–1.53)	1.14 (0.69–1.87)	0.56
Stomach cardia	75	0.98 (0.79–1.23)	1	0.77 (0.36–1.61)	0.75 (0.35–1.61)	0.80 (0.36–1.77)	0.61
Stomach non-cardia	48	1.17 (0.90–1.54)	1	1.37 (0.48–3.90)	1.53 (0.53–4.37)	1.71 (0.56–5.20)	0.35
**Small intestine**	77	1.20 (0.98–1.48)	1	1.13 (0.54–2.38)	1.55 (0.74–3.24)	1.63 (0.71–3.75)	0.17
**Colorectal**	1670	1.02 (0.97–1.06)	1	1.03 (0.89–1.20)	1.01 (0.86–1.18)	1.05 (0.89–1.24)	0.59
Colon	1091	1.04 (0.99–1.10)	1	1.05 (0.87–1.27)	1.14 (0.94–1.38)	1.15 (0.93–1.41)	0.12
Rectum	579	0.97 (0.89–1.05)	1	1.01 (0.79–1.29)	0.80 (0.61–1.05)	0.89 (0.67–1.18)	0.20
**Anal**	60	0.98 (0.77–1.25)	1	0.64 (0.28–1.43)	0.73 (0.31–1.69)	1.09 (0.50–2.40)	0.71
**Hepatobiliary tract**	243	1.03 (0.92–1.16)	1	0.73 (0.47–1.12)	0.94 (0.62–1.42)	0.94 (0.60–1.47)	0.86
**Liver**	157	1.03 (0.89–1.20)	1	0.69 (0.40–1.17)	0.68 (0.40–1.15)	1.00 (0.58–1.72)	0.96
Hepatocellular carcinoma	74	1.11 (0.90–1.37)	1	1.01 (0.45–2.24)	0.51 (0.20–1.26)	1.31 (0.57–3.02)	0.69
Intrahepatic bile duct	67	0.99 (0.77–1.27)	1	0.44 (0.18–1.07)	0.88 (0.41–1.87)	0.84 (0.36–1.97)	0.94
**Pancreas**	386	1.00 (0.91–1.10)	1	0.70 (0.51–0.97) c	0.92 (0.67–1.26)	1.02 (0.73–1.43)	0.61
**Lung**	935	1.05 (0.99–1.11)	1	1.03 (0.83–1.27)	0.90 (0.72–1.13)	1.25 (1.00–1.57)^c^	0.11
**Melanoma skin**	974	1.00 (0.94–1.06)	1	0.89 (0.73–1.09)	0.97 (0.80–1.19)	0.98 (0.79–1.21)	0.94
**Kidney**	451	1.05 (0.97–1.14)	1	1.11 (0.83–1.50)	1.00 (0.73–1.37)	1.25 (0.90–1.72)	0.27
Kidney, except renal pelvis	413	1.06 (0.97–1.15)	1	1.09 (0.80–1.49)	1.03 (0.74–1.44)	1.26 (0.90–1.76)	0.23
Renal cell carcinoma	130	1.08 (0.94–1.24)	1	1.52 (0.83–2.78)	1.43 (0.77–2.67)	1.43 (0.76–2.70)	0.39
**Bladder**	320	1.01 (0.91–1.13)	1	1.24 (0.87–1.77)	1.19 (0.81–1.73)	1.18 (0.79–1.77)	0.49
**Brain and central nervous system**	284	1.08 (0.97–1.20)	1	0.85 (0.59–1.23)	0.82 (0.56–1.20)	1.50 (1.03–2.18) c	0.06
Brain	277	1.09 (0.98–1.21)	1	0.86 (0.59–1.24)	0.82 (0.56–1.21)	1.52 (1.04–2.23)^c^	0.05
Glioma	242	1.06 (0.94–1.19)	1	0.89 (0.60–1.32)	0.79 (0.52–1.19)	1.34 (0.88–2.01)	0.26
**Thyroid**	126	1.11 (0.95–1.29)	1	1.30 (0.70–2.38)	1.32 (0.72–2.44)	1.59 (0.85–3.00)	0.17
**Lymphatic and haematopoietic tissue**	1429	1.01 (0.96–1.06)	1	1.12 (0.95–1.31)	1.06 (0.89–1.25)	1.06 (0.88–1.27)	0.68
**Non-Hodgkin lymphoma**	1091	0.99 (0.94–1.05)	1	1.03 (0.86–1.24)	1.00 (0.82–1.21)	1.00 (0.82–1.23)	0.96
Diffuse large B-cell lymphoma	210	1.04 (0.92–1.17)	1	1.40 (0.88–2.23)	1.48 (0.92–2.38)	1.63 (1.00–2.66) c	0.06
Follicular lymphoma	154	0.89 (0.75–1.05)	1	0.78 (0.48–1.26)	0.75 (0.46–1.25)	0.66 (0.37–1.16)	0.16
CLL/SLL	226	1.03 (0.91–1.16)	1	1.01 (0.68–1.52)	1.18 (0.78–1.78)	1.06 (0.68–1.67)	0.61
**Multiple myeloma**	286	0.98 (0.88–1.10)	1	1.14 (0.79–1.66)	0.93 (0.63–1.37)	0.93 (0.61–1.40)	0.48
**Leukaemia**	400	1.01 (0.92–1.11)	1	1.09 (0.81–1.48)	1.21 (0.88–1.66)	1.07 (0.76–1.51)	0.55
**Breast**^b^	3030	1.00 (0.97–1.03)	1	0.97 (0.87–1.08)	0.97 (0.86–1.08)	1.03 (0.91–1.16)	0.63
Pre-menopausal breast^b^	717	1.00 (0.94–1.07)	1	1.05 (0.81–1.36)	0.98 (0.76–1.27)	1.11 (0.86–1.44)	0.49
Post-menopausal breast^b^	1856	1.00 (0.95–1.04)	1	0.89 (0.77–1.02)	0.94 (0.81–1.08)	1.01 (0.87–1.18)	0.76
**Uterus**^b^	439	1.02 (0.94–1.11)	1	0.87 (0.64–1.17)	0.92 (0.68–1.24)	1.04 (0.76–1.41)	0.74
Endometrium^b^	429	1.03 (0.95–1.12)	1	0.88 (0.65–1.20)	0.93 (0.69–1.26)	1.06 (0.77–1.45)	0.65
**Ovary**^b^	291	1.19 (1.08–1.30)^e^, ^f^	1	0.96 (0.65–1.41)	1.15 (0.78–1.69)	1.45 (0.98–2.15)	0.03
**Prostate**^b^	3621	0.99 (0.96–1.02)	1	1.04 (0.94–1.15)	1.05 (0.95–1.17)	1.05 (0.93–1.17)	0.39

UPF, ultra-processed food; HR, hazard ratio; CI, confidence interval; Ref, reference category; CLL, Chronic lymphocytic leukemia; SLL, Small lymphocytic lymphoma.

All models were fully adjusted with age (underlying timescale), ethnicity, smoking status, physical activity level, average household income, highest educational attainment, alcohol intake, body mass index, total daily energy intake, and stratified by sex, height, family history of cancer, index of multiple deprivation quintile, and geographical region. Analyses of female-specific cancers were additionally adjusted for baseline menopausal status, use of oral contraceptives, use of hormone replacement therapy, and parity.

a UPF consumption was defined as the percentage of its weight contribution relative to total food intake measured in g/day. Study participants were further categorized into quartiles (Q1-Q4 represents lowest to highest quartile of UPF consumption).

b Modelling for breast, uterus and ovarian cancers were conducted in women only (n = 107919), modelling for prostate cancer were conducted in men only (n = 89507).

c *P* < 0.05.

d *P* < 0.01.

e *P* < 0.001.

f Bonferroni-corrected *P* < 0.05.

Furthermore, when analysing UPF consumption quartiles (Table 2), participants with the highest compared with lowest UPF quartile had a higher risk of overall cancer by 7% (95% CI, 1.02–1.14), lung cancer by 25% (95% CI, 1.01–1.57), brain cancer by 52% (95% CI, 1.04–2.23), and diffuse large B-cell lymphoma by 63% (95% CI, 1.00–2.66). Conversely, a significantly lower risk of head and neck cancer was observed among those with higher UPF quartile (e.g. HR, 0.59; 95% CI, 0.41–0.85 for the highest compared with lowest UPF quartile). Similarly, stratified analyses showed lower risk patterns for head and neck cancer among never smokers, ex-smokers and all alcohol consumption groups, but most findings did not reach statistical significance (Appendix Table S4).

### UPF consumption and cancer mortality

A total of 4009 cancer deaths occurred during the 1,958,878 person-years of follow-up (median, 9.8 years; IQR, 9.5–10.7 years). Hazard ratios from minimally to fully adjusted models mostly reflect a higher mortality of overall and site-specific cancers with increasing levels of UPF consumption (Appendix Fig. S3). In fully adjusted models (Table 3), associations persisted for overall cancer mortality (HR, 1.06; 95% CI, 1.03–1.09), breast cancer mortality in females (HR, 1.16; 95% CI, 1.02–1.32), and ovarian cancer mortality (HR, 1.30; 95% CI, 1.13–1.50) per 10 percentage points increment in UPF consumption. Moreover, overall cancer and ovarian cancer mortality results remained statistically significant after Bonferroni correction (Table 3). Participants with the highest compared with lowest UPF quartile had a higher risk of overall (HR, 1.17; 95% CI, 1.05–1.30), lung (HR, 1.38; 95% CI, 1.04–3.82), and ovarian (HR, 1.91; 95% CI, 1.08–3.39) cancer mortality. These associations remained broadly similar in sensitivity analyses except for lung cancer mortality (Appendix Table S5).

**Table 3:** Association between ultra-processed food consumption in the total diet and cancer-related mortality.

Cancer site	Number of deaths	per 10% increment in UPF intake^a^ HR (95% CI)	Quartile of UPF consumption^a^				*P*_Trend_
			Q1 (lowest)	Q2	Q3	Q4 (highest)	
			Ref	HR (95% CI)	HR (95% CI)	HR (95% CI)	
**All cancers**	4009	1.06 (1.03–1.09)^e^, ^f^	1	1.00 (0.91–1.10)	1.00 (0.91–1.11)	1.17 (1.05–1.30)^d^	0.006
**Head and Neck**	54	0.95 (0.71–1.27)	1	0.53 (0.20–1.41)	0.95 (0.40–2.22)	0.60 (0.21–1.70)	0.61
**Gastrointestinal**	1408	1.04 (0.99–1.09)	1	0.89 (0.75–1.05)	1.01 (0.85–1.19)	1.04 (0.87–1.25)	0.37
**Oesophagus**	194	1.08 (0.95–1.24)	1	1.27 (0.81–1.99)	0.98 (0.60–1.60)	1.31 (0.79–2.16)	0.52
**Stomach**	121	1.16 (0.99–1.35)	1	0.98 (0.52–1.85)	1.04 (0.56–1.93)	1.24 (0.65–2.35)	0.45
**Colorectal**	438	1.03 (0.94–1.12)	1	0.94 (0.70–1.27)	1.09 (0.80–1.47)	1.01 (0.72–1.41)	0.71
Colon	244	0.98 (0.87–1.11)	1	1.05 (0.70–1.59)	1.21 (0.79–1.84)	1.07 (0.68–1.68)	0.64
Rectum	194	1.08 (0.95–1.22)	1	0.85 (0.55–1.33)	0.95 (0.61–1.49)	0.97 (0.59–1.59)	0.99
**Hepatobiliary tract**	182	1.04 (0.91–1.19)	1	0.78 (0.47–1.28)	1.01 (0.62–1.65)	0.93 (0.55–1.58)	0.92
**Liver**	150	1.03 (0.88–1.20)	1	0.61 (0.35–1.07)	0.98 (0.58–1.65)	0.91 (0.51–1.61)	0.87
Hepatocellular carcinoma	46	1.12 (0.85–1.49)	1	0.52 (0.17–1.56)	0.46 (0.15–1.38)	0.85 (0.29–2.50)	0.74
Intrahepatic bile duct	103	1.01 (0.84–1.22)	1	0.71 (0.36–1.41)	1.34 (0.71–2.50)	1.00 (0.50–2.03)	0.54
**Pancreas**	371	1.00 (0.90–1.10)	1	0.74 (0.53–1.04)	0.92 (0.67–1.27)	1.04 (0.73–1.47)	0.61
**Lung**	633	1.06 (0.99–1.14)	1	1.20 (0.92–1.55)	1.06 (0.81–1.40)	1.38 (1.04–1.82)^c^	0.07
**Melanoma skin**	63	1.15 (0.89–1.49)	1	1.26 (0.55–2.84)	1.68 (0.73–3.84)	1.13 (0.43–2.97)	0.59
**Kidney**	117	1.04 (0.89–1.22)	1	1.59 (0.90–2.80)	1.07 (0.57–1.99)	1.35 (0.72–2.53)	0.64
**Bladder**	112	1.05 (0.87–1.25)	1	0.93 (0.50–1.72)	1.05 (0.56–1.98)	1.19 (0.61–2.34)	0.55
**Brain and central nervous system**	251	1.05 (0.93–1.18)	1	0.78 (0.53–1.15)	0.77 (0.51–1.15)	1.26 (0.84–1.89)	0.34
**Lymphatic and haematopoietic tissue**	376	1.05 (0.95–1.15)	1	1.24 (0.89–1.71)	0.88 (0.62–1.26)	1.21 (0.85–1.74)	0.70
**Non-Hodgkin lymphoma**	141	1.02 (0.87–1.20)	1	1.22 (0.73–2.03)	0.63 (0.34–1.18)	1.12 (0.62–2.03)	0.78
**Multiple myeloma**	91	1.08 (0.89–1.31)	1	1.09 (0.53–2.24)	0.75 (0.35–1.59)	1.01 (0.47–2.17)	0.79
**Leukaemia**	137	1.02 (0.87–1.19)	1	1.33 (0.77–2.32)	1.38 (0.77–2.46)	1.32 (0.71–2.47)	0.40
**Breast**^b^	176	1.16 (1.02–1.32)^c^	1	1.13 (0.70–1.81)	1.30 (0.80–2.12)	1.62 (0.98–2.68)	0.05
Post-menopausal^b^	120	1.11 (0.94–1.31)	1	0.72 (0.39–1.29)	0.94 (0.51–1.73)	1.24 (0.67–2.31)	0.41
**Uterus**^b^	61	1.01 (0.76–1.33)	1	0.85 (0.38–1.91)	0.96 (0.42–2.19)	0.78 (0.30–2.01)	0.71
Endometrium^b^	43	0.99 (0.70–1.40)	1	0.67 (0.23–1.96)	1.05 (0.38–2.93)	0.80 (0.24–2.58)	0.93
**Ovary**^b^	143	1.30 (1.13–1.50)^e^, ^f^	1	0.82 (0.45–1.49)	1.56 (0.89–2.73)	1.91 (1.08–3.39)^c^	0.005
**Prostate**^b^	194	0.98 (0.85–1.12)	1	0.95 (0.61–1.49)	0.91 (0.56–1.47)	0.92 (0.56–1.53)	0.74

UPF, ultra-processed food; HR, hazard ratio; CI, confidence interval; Ref, reference category.

All models were fully adjusted with age (underlying timescale), ethnicity, smoking status, physical activity level, average household income, highest educational attainment, alcohol intake, body mass index, total daily energy intake, and stratified by sex, height, family history of cancer, index of multiple deprivation quintile, and geographical region. Analyses of female-specific cancers were additionally adjusted for baseline menopausal status, use of oral contraceptives, use of hormone replacement therapy, and parity.

a UPF consumption was defined as the percentage of its weight contribution relative to total food intake measured in g/day. Study participants were further categorized into quartiles (Q1-Q4 represents lowest to highest quartile of UPF consumption).

b Modelling for breast, uterus and ovarian cancers were conducted in women only (n = 107919), modelling for prostate cancer were conducted in men only (n = 89507).

c *P* < 0.05.

d *P* < 0.01.

e *P* < 0.001.

f Bonferroni-corrected *P* < 0.05.

### Calorie contribution of UPFs in diet and cancer risk

The mean calorie contribution of UPFs was 48.6% (SD, 15.8%) of total energy intake (kcal/day) and ranged from 28.4% (7.6%) to 68.7% (7.5%) for the lowest to highest UPF quartiles (Appendix Fig. S4). The fully adjusted results suggest a positive association between the proportion of daily calories sourced from UPFs and incidence of overall cancer, cancers of lymphatic and haematopoietic tissues, and ovarian cancer (Appendix Table S6). Evaluation of cancer mortality showed significantly positive associations for overall, oesophagus, and ovarian cancers (Appendix Table S7).

## Discussion

This large prospective cohort analysis conducted within the UK Biobank provides a comprehensive assessment of associations between UPF consumption and risk of many site-specific cancer outcomes for the first time to our knowledge. There are three particularly noteworthy findings: First, every 10% increment in UPF content of diet was associated with an increased incidence of overall cancer by 2% and ovarian cancer by 19%. Second, participants with the highest compared with lowest UPF consumption quartile had higher incidence of overall and brain cancer, and a lower incidence of head and neck cancer. Finally, every 10% increment in UPF consumption was associated with increased mortality of overall cancer by 6%, breast cancer by 16%, and ovarian cancer by 30%. These associations persisted after adjustment for a range of key socio-economic, behavioural and dietary factors.

No previously published study has assessed the incidence and mortality comprehensively for site-specific cancers in relation to UPF consumption. Only two previously published studies assessed incidence of common cancer sites.^8^ The French NutriNet-Santé study showed a 12% and 11% increase in risk of overall and breast cancer per 10% increment in UPF consumption (g/day), and no evidence of association for prostate and colorectal cancer.^8^ A recent study from the US found a 29% increase in the risk of colorectal cancer among men in the highest quintile of UPF consumers compared with the lowest (based on servings/day).^9^ Our findings are more aligned with the NutriNet-Santé study for overall, prostate, and colorectal cancer incidence, but not for breast cancer incidence. While we used the same measure of UPF consumption (weight ratio) as the NutriNet-Santé study, the US study had a much longer follow-up time (24–28 years compared with 5 years in the NutriNet-Santé and 10 years in this study). Moreover, our study cohort had a larger proportion of never smokers than in previous studies and these differences in characteristics, design and setting may all contribute to the differences in findings. Mortality of site-specific cancers have not been previously assessed, but overall cancer mortality has been examined in three cohort studies conducted in Spain, Italy, and North America with similar lengths of follow-up to this study.^10^, ^11^, ^12^ None of the three studies, however, have identified any significant association with cancer mortality. Our study cohort is more than two-folds larger than previous studies and comparatively older due to the recruitment of middle-aged adults. Moreover, our study cohort was originated from a population with a substantially greater consumption of UPFs. Importantly, our study presents findings for many less common cancers not examined before, and our findings of the positive associations between UPF consumption and risks and associated mortality of overall and ovarian cancer were consistent among weight and energy ratio measures of UPF consumption.

Various mechanisms may explain the positive associations found between UPF consumption and the risk of adverse cancer outcomes. Recommendations for cancer prevention emphasise the importance of nutritionally balanced diets involving greater consumption of vegetables and fruit, lower consumption of unprocessed red meat and avoidance of processed meat, besides other behavioural factors including alcohol consumption and smoking.^2^^,^^4^ However, dietary patterns with a high UPF content are generally nutritionally inferior and are higher in energy, total and saturated fats, salt, and free sugars, and lower in fibre and several micronutrients.^5^ Alteration of food matrices by ultra-processing results in degradation of food health potential and deterioration of nutrient bioavailability and bioaccessibility.^19^ Furthermore, evidence has been accumulating on the strong obesity and T2D-promoting potential of UPFs,^7^ both of which are risk factors for many cancers including those of the digestive tract and some hormone-related cancers in women.^4^^,^^20^^,^^21^

Emerging research has suggested other common properties of UPFs that may contribute to adverse cancer outcomes, including through the use of controversial food additives, neoformed contaminants during ultra-processing, and toxic contaminants migrated from food packaging. Recent evidence from the NutriNet-Santé cohort showed higher intake of artificial sweeteners associated with increased risk of overall, breast, and obesity-related cancers,^13^ while higher intake of nitrate and nitrite from food additives was associated with increased risk of breast and prostate cancer, respectively.^22^ Higher dietary exposure of acrylamide, an industrial chemical formed during high-temperature cooking procedures, was found associated with an increased risk of ovarian and endometrial cancers.^23^ Phthalates and bisphenols are endocrine-disrupting chemicals commonly found in food storage, packaging and contacting materials, and higher urinary concentration of phthalates and bisphenols-F (analogue of the more regulated bisphenol-A) have been detected in individuals with higher UPF consumption.^24^ Available data on bisphenols are predominantly experimental but have consistently shown many toxic effects including for human breast cancer and damage to DNA, nervous and immune systems.^14^ Epidemiological studies have suggested a positive association between phthalates and T2D and insulin resistance.^25^^,^^26^ Recent animal models have shown that phthalates may induce neuroinflammation and disruption of the blood–brain barrier.^27^ No previous studies have assessed the link between UPF consumption and brain cancer, but human studies have demonstrated associations with potential harmful effects on brain functions.^28^^,^^29^

This study has many strengths. The large sample size and long prospective follow-up has enabled the investigation into outcomes of many anatomical cancer sites, especially site-specific cancer mortality. Also, the date and type of cancer incidence and mortality were ascertained through national cancer and death registries. The use of validated 24-h recalls allowed for more detailed dietary data being captured than many food frequency questionnaires, crucial for food processing classifications.^17^

Our study has important limitations. First, the study cohort was not nationally representative and may over-represent populations with white ethnicity and those living in a less socio-economically deprived areas, and the mean UPF consumption and prevalence of obesity were lower than the UK average.^30^ However, this study has reported important associations comparing cancer risk and mortality by levels of UPF consumption which may still be generalisable to the wider population or similar cohorts in other contexts. Second, misclassification of a few food items may occur owing to limited food processing information and we assigned them to the most probable food group based on published findings of common foods and drinks consumed in the UK.^30^ Third, while we considered the average of multiple 24-h recalls the best representation of usual dietary intake, 39.7% of the cohort had only one 24-h recall and may be prone to measurement error owing to its limited ability to fully capture individuals’ variation in diet. Fourth, the associations for head and neck cancer incidence may be partly due to the complex interrelationships between UPF consumption, alcohol intake and smoking. However, we could not explore these further due to limited sample size shown in exploratory analysis. Finally, although we adjusted the analyses for a wide range of potential confounders including key lifestyle and nutritional factors, residual confounding may have affected the findings due to observational nature of the study.

In summary, this large contemporary prospective study of middle-aged UK adults found that higher UPF consumption was associated with a greater incidence and mortality of overall and certain site-specific cancers. Although causality may not be implied owing to the observational nature of the study, these findings highlight the importance of considering degrees of food processing in diets. In particular, the associations were found most consistent for overall cancer and ovarian cancer outcomes in women. These findings suggest that limiting UPF consumption may be beneficial to prevent and reduce the modifiable burdens of cancer.

## Contributors

KC, CM, and EPV conceptualised the study and all authors contributed to the study design. KC compiled the data and performed statistical analyses with supervisory input from EPV. KC and EPV had access and verified the underlying data used in this study. All authors contributed to the finalisation of statistical models and interpretation of findings. KC and EPV wrote the first draft of the manuscript, and MJG, FR, RBL, IH, NK, and CM critically reviewed and edited the manuscript. All authors had full access to all the data in the study, approved the final manuscript, and accept responsibility for the decision to submit for publication.

## Data sharing statement

UK Biobank data are available through application to the database https://www.ukbiobank.ac.uk/.

## Declaration of interests

All authors declare no conflict of interest.

## Appendix A Supplementary data

UKB-UPF-Cancer-Appendix-Revised-FINAL
