# Supplementary material for: Ultra-processed food consumption, cancer risk and cancer mortality: a large-scale prospective analysis within the UK Biobank
Source: eClinicalMedicine. 2023 Jan 31;56:101840. doi: 10.1016/j.eclinm.2023.101840 (PMC9985039; doi:10.1016/j.eclinm.2023.101840)
Supplement: UKB-UPF-Cancer-Appendix-Revised-FINAL [file mmc1.pdf]

Appendix

Ultra-processed food consumption, cancer risk and cancer mortality: a large-scale prospective analysis within the UK Biobank

Kiara Chang, Marc J. Gunter, Fernanda Rauber, Renata B. Levy, Inge Huybrechts, Nathalie Kliemann, Christopher Millett, Eszter P. Vamos

Table S1: Coding and number of site-specific cancer incident cases among UK Biobank participants 2

Table S2: Coding and number of site-specific cancer-related deaths among UK Biobank participants 4

Figure S1: Flow diagram for the derivation of study cohort 5

Figure S2: Covariate adjustments for the association between UPF consumption (per 10 percentage points) and cancer incidence 6

Table S3: Sensitivity analyses for the association between UPF consumption (per 10 percentage points) and cancer incidence 7

Table S4: Results of stratified analysis for head and neck and lung cancer outcomes 9

Figure S3: Covariate adjustments for the association between UPF consumption (per 10 percentage points) and cancer-related mortality 10

Table S5: Sensitivity analyses for the association between UPF consumption (per 10 percentage points) and cancer-related mortality 11

Figure S4: Sources of NOVA subgroups based on total energy intake by quartile of UPF consumption 13

Table S6: Association of cancer incidence by levels of UPF consumption based on total energy intake 14

Table S7: Association of cancer-related mortality by levels of UPF consumption based on total energy intake 16

Table S1: Coding and number of site-specific cancer incident cases among UK Biobank participants

Cancer site	ICD-10	ICD-O-3 morphology codes	Number of incident cases	Cases per 1000 person-years
**All cancers (excl. C44 non-melanoma skin cancer)**	C00-C97		15921	8·42
**Head and Neck**	C00-C14, C30-C32		342	0·18
Oral cavity	C02-C06		106	0·06
Oropharynx	C09-C10		86	0·05
Hypopharynx	C13		4	0·002
Larynx	C32		33	0·02
**Gastrointestinal**	C15-C26		2937	1·55
**Oesophagus**	C15		283	0·15
Adenocarcinoma	C15	8140, 8141, 8143, 8144, 8145, 8190, 8200, 8201, 8210, 8211, 8213, 8214, 8215, 8220, 8221, 8230, 8231, 8260, 8261, 8262, 8263, 8310, 8410, 8480, 8481, 8482, 8490, 8550, 8551, 8570, 8571, 8572, 8573, 8574, 8576	186	0·10
Squamous cell carcinoma (SCC)	C15	8050, 8052, 8070, 8071, 8072, 8073, 8074, 8075, 8076, 8078, 8083, 8084	67	0·04
**Stomach**	C16		189	0·10
Stomach cardia	C160		75	0·04
Stomach non-cardia	C161-C166		48	0·03
**Small intestine**	C17		77	0·04
**Colorectal**	C18-C20		1670	0·88
Colon	C18		1091	0·58
Rectum	C19-C20		579	0·31
**Anal**	C21		60	0·03
**Hepatobiliary tract**	C22-C24		243	0·13
**Liver**	C22		157	0·08
Hepatocellular carcinoma (HCC)	C220	8170, 8171, 8172, 8173, 8174, 8175	74	0·04
Intrahepatic bile duct	C221		67	0·04
**Pancreas**	C25	Any except 8150, 8151, 8153, 8155, 8240, 8246, 9591	386	0·20
**Lung (including trachea and bronchus)**	C33-C34		935	0·49
**Melanoma skin**	C43	8720, 8721, 8722, 8723, 8728, 8730, 8740, 8741, 8742, 8743, 8744, 8745, 8746, 8761, 8770, 8772, 8773, 8774, 8780	974	0·52
**Kidney**	C64-C65		451	0·24
Kidney, except renal pelvis	C64		413	0·22
Renal pelvis	C65		38	0·02
Renal cell carcinoma (RCC)	C64	8312	130	0·07
**Bladder**	C67		320	0·17

Table S1 (continued)

Cancer site	ICD-10	ICD-O-3 morphology codes	Number of incident cases	Cases per 1000 person-years
**Brain and central nervous system**	C70-C72		284	0·15
Brain	C71		277	0·15
Glioma	C71	9380, 9381, 9382, 9390, 9391, 9392, 9393, 9395, 9400, 9401, 9411, 9420, 9423, 9424, 9425, 9430, 9440, 9441, 9442, 9450, 9451, 9460, 9470, 9471, 9472, 9473, 9474, 9480	242	0·13
**Thyroid**	C73		126	0·07
**Lymphatic and haematopoietic tissue**	C81–C96		1429	0·76
**Non-Hodgkin lymphoma**		9590, 9591, 9596, 9597, 9670, 9671, 9673, 9675, 9678, 9679, 9680, 9684, 9687, 9688, 9689, 9690, 9691, 9695, 9698, 9699, 9700, 9701, 9702, 9705, 9708, 9709, 9712, 9714, 9716, 9717, 9718, 9719, 9726, 9727, 9728, 9729, 9731, 9732, 9733, 9734, 9735, 9737, 9738, 9760, 9761, 9764, 9820, 9823, 9826, 9827, 9831, 9832, 9833, 9834, 9835, 9836, 9837, 9940, 9948	1091	0·58
Diffuse large B-cell lymphoma (DLBCL)		9678, 9679, 9680, 9684, 9688, 9712, 9735, 9737, 9738	210	0·11
Follicular lymphoma (FL)		9690, 9691, 9695, 9698	154	0·08
Chronic lymphocytic leukemia/Small lymphocytic lymphoma (CLL/SLL)		9670, 9823	226	0·12
Marginal-zone lymphoma (MZL)		9689, 9699, 9760, 9764	1	0·001
**Multiple myeloma**	C90		286	0·15
**Leukaemia**	C91-C95		400	0·21
**Breast**	C50		3053	2·93
Pre-menopausal breast	C50		717	0·69
Post-menopausal breast	C50		1856	1·78
**Cervical**	C53		36	0·03
**Uterus**	C54-C55		439	0·42
Endometrium	C54		429	0·41
**Ovary**	C56		291	0·28
**Prostate**	C61		3621	4·27

Table S2: Coding and number of site-specific cancer-related deaths among UK Biobank participants

Cancer site	ICD-10	Number of deaths	Deaths per 1000 person-years
**All cancers (excl. C44 non-melanoma skin cancer)**	C00-C97	4009	2·05
**Head and Neck**	C00-C14, C30-C32	54	0·03
Oral cavity	C02-C06	18	0·01
Oropharynx	C09-C10	11	0·01
Hypopharynx	C13	4	0·002
Larynx	C32	8	0·004
**Gastrointestinal**	C15-C26	1408	0·72
**Oesophagus**	C15	194	0·10
**Stomach**	C16	121	0·06
Stomach cardia	C160	24	0·01
Stomach non-cardia	C161-C166	1	0·001
**Small intestine**	C17	20	0·01
**Colorectal**	C18-C20	438	0·22
Colon	C18	244	0·12
Rectum	C19-C20	194	0·10
**Anal**	C21	11	0·01
**Hepatobiliary tract**	C22-C24	182	0·09
**Liver**	C22	150	0·08
Hepatocellular carcinoma (HCC)	C220	46	0·02
Intrahepatic bile duct	C221	103	0·05
**Pancreas**	C25	371	0·19
**Lung (including trachea and bronchus)**	C33-C34	633	0·32
**Melanoma skin**	C43	63	0·03
**Kidney**	C64-C65	117	0·06
Kidney, except renal pelvis	C64	115	0·06
Renal pelvis	C65	2	0·001
**Bladder**	C67	112	0·06
**Brain and central nervous system**	C70-C72	251	0·13
Brain	C71	250	0·13
**Thyroid**	C73	8	0·004
**Lymphatic & haematopoietic tissue**	C81–C96	376	0·19
**Non-Hodgkin lymphoma**	C82-C85	141	0·07
**Multiple myeloma**	C90	91	0·05
**Leukaemia**	C91-C95	137	0·07
**Breast**	C50	176	0·16
Pre-menopausal breast	C50	30	0·03
Post-menopausal breast	C50	120	0·11
**Cervical**	C53	8	0·01
**Uterus**	C54-C55	61	0·06
Endometrium	C54	43	0·04
**Ovary**	C56	143	0·13
**Prostate**	C61	194	0·22

Figure S1: Flow diagram for the derivation of study cohort

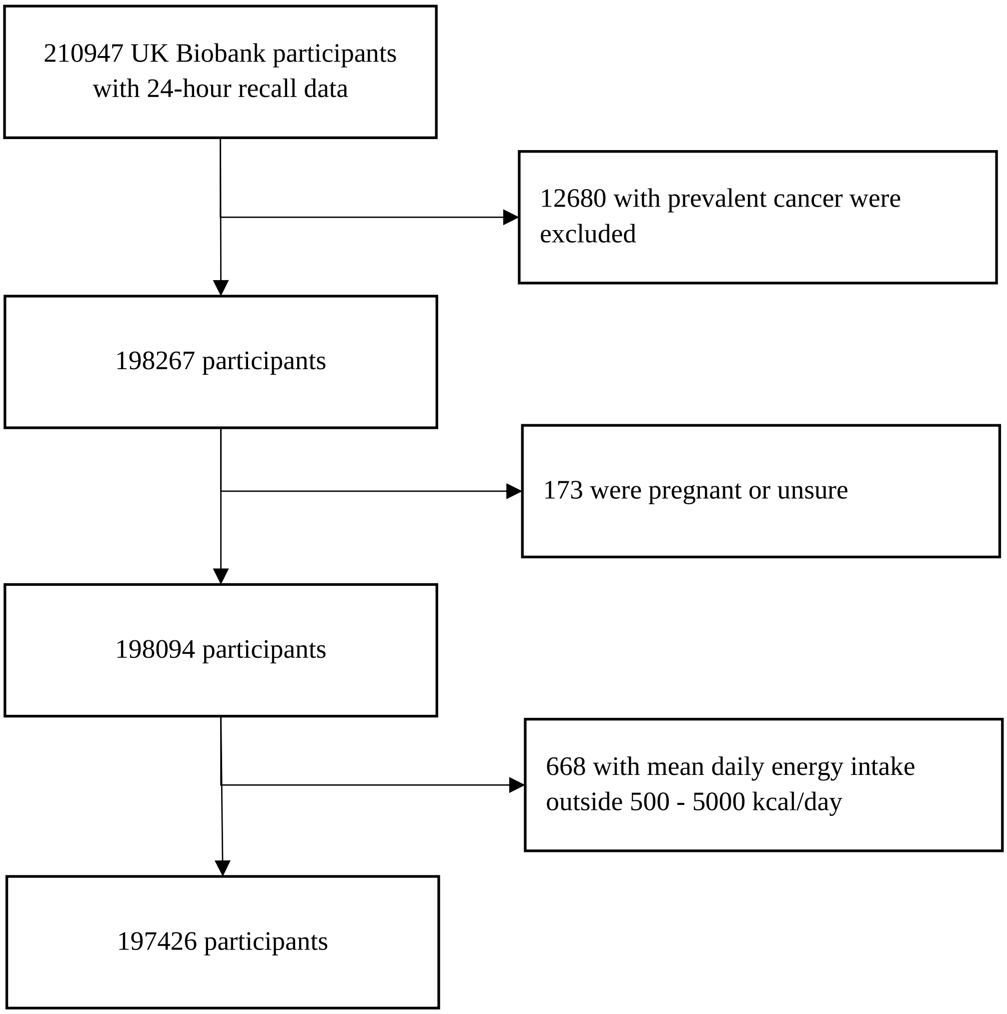

Figure S2: Covariate adjustments for the association between UPF consumption (per 10 percentage points) and cancer incidence

Cancer site	Number of incident cases	Model 1 Hazard Ratio (95% CI)	Model 2 Hazard Ratio (95% CI)	Model 3 Hazard Ratio (95% CI)	Model 4 Hazard Ratio (95% CI)	Model 4 P value
**All cancers**	15921	**1.03 (1.02, 1.04)**	**1.03 (1.02, 1.04)**	**1.02 (1.01, 1.04)**	**1.02 (1.01, 1.04)**	**0.005**
**Head and Neck**	342	0.91 (0.84, 1.00)	0.91 (0.82, 1.00)	0.90 (0.82, 1.00)	0.89 (0.80, 1.00)	0.05
- Oral cavity	106	0.96 (0.82, 1.12)	0.95 (0.80, 1.12)	0.95 (0.81, 1.12)	0.91 (0.76, 1.08)	0.30
- Oropharynx	86	0.89 (0.75, 1.06)	0.94 (0.76, 1.15)	0.93 (0.76, 1.15)	0.89 (0.71, 1.11)	0.31
**Gastrointestinal**	2937	**1.04 (1.01, 1.07)**	**1.04 (1.01, 1.07)**	1.02 (0.99, 1.05)	**1.03 (1.00, 1.07)**	**0.04**
**Oesophagus**	283	1.04 (0.95, 1.14)	1.07 (0.97, 1.19)	1.05 (0.95, 1.17)	1.08 (0.97, 1.21)	0.12
- Adenocarcinoma	186	1.03 (0.92, 1.15)	1.05 (0.92, 1.20)	1.02 (0.89, 1.16)	1.05 (0.91, 1.20)	0.48
- Squamous cell carcinoma	67	0.91 (0.74, 1.12)	0.93 (0.74, 1.18)	0.94 (0.74, 1.21)	0.96 (0.74, 1.24)	0.77
**Stomach**	189	**1.14 (1.03, 1.26)**	1.10 (0.97, 1.24)	1.09 (0.96, 1.23)	1.08 (0.95, 1.22)	0.219
- Stomach cardia	75	1.07 (0.91, 1.27)	1.06 (0.87, 1.30)	1.05 (0.85, 1.28)	0.98 (0.79, 1.23)	0.921
- Stomach non-cardia	48	**1.24 (1.03, 1.50)**	1.19 (0.93, 1.53)	1.20 (0.93, 1.56)	1.17 (0.90, 1.54)	0.23
**Small intestine**	77	1.12 (0.96, 1.32)	1.18 (0.97, 1.44)	1.18 (0.97, 1.44)	1.20 (0.98, 1.48)	0.071
**Colorectal**	1670	1.01 (0.97, 1.05)	1.01 (0.97, 1.06)	1.01 (0.96, 1.05)	1.02 (0.97, 1.06)	0.34
- Colon	1091	1.04 (0.99, 1.09)	1.04 (0.99, 1.10)	1.04 (0.98, 1.09)	1.04 (0.99, 1.10)	0.09
- Rectum	579	0.95 (0.89, 1.01)	0.95 (0.88, 1.03)	0.95 (0.88, 1.02)	0.97 (0.89, 1.05)	0.45
**Anal**	60	0.97 (0.79, 1.19)	0.95 (0.75, 1.20)	0.96 (0.76, 1.21)	0.98 (0.77, 1.25)	0.88
**Hepatobiliary tract**	243	**1.15 (1.05, 1.26)**	1.07 (0.96, 1.20)	1.03 (0.92, 1.16)	1.03 (0.92, 1.16)	0.55
**Liver**	157	**1.14 (1.02, 1.28)**	1.09 (0.94, 1.25)	1.04 (0.90, 1.20)	1.03 (0.89, 1.20)	0.60
- Hepatocellular carcinoma	74	**1.27 (1.09, 1.47)**	1.20 (0.98, 1.46)	1.14 (0.93, 1.39)	1.11 (0.90, 1.37)	0.32
- Intrahepatic bile duct	67	1.00 (0.83, 1.21)	0.98 (0.76, 1.25)	0.96 (0.75, 1.23)	0.99 (0.77, 1.27)	0.98
**Pancreas**	386	1.00 (0.92, 1.08)	1.02 (0.93, 1.11)	1.00 (0.91, 1.09)	1.00 (0.91, 1.10)	0.91
**Lung**	935	**1.11 (1.05, 1.16)**	1.04 (0.98, 1.10)	1.04 (0.98, 1.10)	1.05 (0.99, 1.11)	0.07
**Melanoma skin**	974	0.99 (0.94, 1.04)	1.01 (0.96, 1.07)	1.01 (0.95, 1.07)	1.00 (0.94, 1.06)	0.88
**Kidney**	451	**1.11 (1.03, 1.18)**	1.05 (0.98, 1.14)	1.03 (0.95, 1.11)	1.05 (0.97, 1.14)	0.18
- Kidney, except renal pelvis	413	**1.11 (1.03, 1.19)**	1.07 (0.98, 1.16)	1.04 (0.96, 1.13)	1.06 (0.97, 1.15)	0.13
- Renal cell carcinoma	130	**1.25 (1.12, 1.40)**	**1.14 (1.00, 1.30)**	1.08 (0.94, 1.24)	1.08 (0.94, 1.24)	0.24
**Bladder**	320	1.01 (0.93, 1.10)	1.00 (0.90, 1.11)	0.98 (0.88, 1.09)	1.01 (0.91, 1.13)	0.73
**Brain and central nervous system**	284	1.04 (0.95, 1.14)	1.08 (0.97, 1.19)	1.08 (0.97, 1.19)	1.08 (0.97, 1.20)	0.12
- Brain	277	1.04 (0.95, 1.14)	1.09 (0.98, 1.20)	1.09 (0.98, 1.20)	1.09 (0.98, 1.21)	0?10
- Glioma	242	1.04 (0.94, 1.14)	1.06 (0.95, 1.19)	1.06 (0.95, 1.18)	1.06 (0.94, 1.19)	0.28
**Thyroid**	126	**1.16 (1.03, 1.31)**	1.14 (0.99, 1.32)	1.13 (0.98, 1.30)	1.11 (0.95, 1.29)	0.15
**Lymphatic & haematopoietic tissue**	1429	**1.04 (1.00, 1.09)**	1.04 (0.99, 1.08)	1.03 (0.98, 1.07)	1.01 (0.96, 1.06)	0.68
**Non-Hodgkin lymphoma**	1091	1.04 (0.99, 1.09)	1.03 (0.98, 1.08)	1.02 (0.97, 1.07)	0.99 (0.94, 1.05)	0.95
- Diffuse large B-cell lymphoma	210	**1.11 (1.01, 1.23)**	1.08 (0.97, 1.21)	1.07 (0.96, 1.20)	1.04 (0.92, 1.17)	0.46
- Follicular lymphoma	154	0.94 (0.82, 1.07)	0.93 (0.80, 1.09)	0.92 (0.78, 1.07)	0.89 (0.75, 1.05)	0.19
- CLL/SLL	226	1.02 (0.92, 1.13)	1.02 (0.91, 1.15)	1.01 (0.90, 1.14)	1.03 (0.91, 1.16)	0.63
**Multiple myeloma**	286	1.05 (0.96, 1.15)	1.03 (0.93, 1.14)	1.01 (0.91, 1.13)	0.98 (0.88, 1.10)	0.82
**Leukaemia**	400	1.03 (0.96, 1.11)	1.03 (0.94, 1.12)	1.02 (0.93, 1.11)	1.01 (0.92, 1.11)	0.68
**Breast**	3030	1.01 (0.98, 1.03)	1.01 (0.98, 1.04)	1.00 (0.97, 1.03)	1.00 (0.97, 1.03)	0.91
- Pre-menopausal breast	717	1.00 (0.95, 1.05)	1.01 (0.95, 1.07)	1.01 (0.95, 1.07)	1.00 (0.94, 1.07)	0.78
- Post-menopausal breast	1856	1.02 (0.98, 1.05)	1.02 (0.98, 1.06)	1.01 (0.96, 1.05)	1.00 (0.95, 1.04)	0.92
**Uterus**	439	**1.09 (1.01, 1.16)**	**1.11 (1.03, 1.20)**	1.05 (0.97, 1.13)	1.02 (0.94, 1.11)	0.49
- Endometrium	429	**1.09 (1.02, 1.17)**	**1.12 (1.03, 1.21)**	1.05 (0.97, 1.14)	1.03 (0.95, 1.12)	0.44
**Ovary**	291	**1.19 (1.10, 1.29)**	**1.20 (1.09, 1.32)**	**1.19 (1.08, 1.31)**	**1.19 (1.08, 1.30)**	**<0.001**
**Prostate**	3621	0.99 (0.96, 1.01)	0.99 (0.96, 1.02)	0.99 (0.96, 1.02)	0.99 (0.96, 1.02)	0.74

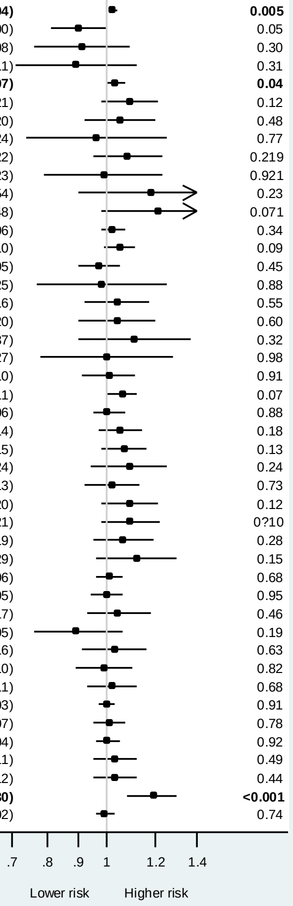

Abbreviations: UPF, ultra-processed food; CI, confidence interval; CLL, Chronic lymphocytic leukemia; SLL, Small lymphocytic lymphoma.

UPF consumption was defined as the percentage of its weight contribution relative to total food intake measured in g/day. Model results are interpreted as hazard ratio for every 10 percentage points increment in the UPF content of total diet. Model 1 included age (underlying timescale), and stratified by sex. Model 2 additionally included ethnicity, smoking status, physical activity level, average household income, highest educational attainment, alcohol intake, and additionally stratified by height, family history of cancer, index of multiple deprivation quintile, and geographical region. Female-specific cancer outcomes were additionally adjusted for baseline menopausal status, use of oral contraceptives, use of hormone replacement therapy, and parity. Model 3 additionally included body mass index category. Model 4 additionally included total daily energy intake. Analysis for risk of breast, uterus and ovarian cancers were conducted in women only (n=107919) and risk of prostate cancer was conducted in men only (n= 89507).

Table S3: Sensitivity analyses for the association between UPF consumption (per 10 percentage points) and cancer incidence

Cancer Site	Model S1 HR (95% CI)	Model S2 HR (95% CI)	Model S3 HR (95% CI)	Model S4 HR (95% CI)	Model S5 HR (95% CI)	Model S6 HR (95% CI)	Model S7 HR (95% CI)
**All cancers**	**1.02 (1.00-1.03)§**	**1.02 (1.00-1.03)‡**	**1.01 (1.00-1.03)‡**	**1.02 (1.00-1.03)§**	**1.01 (1.00-1.03)‡**	**1.02 (1.00-1.03)§**	**1.02 (1.00-1.04)§**
**Head and Neck**	0.89 (0.80-1.00)	0.88 (0.79-1.00)	0.90 (0.81-1.01)	**0.89 (0.80-0.99)‡**	0.89 (0.80-1.00)	0.90 (0.81-1.00)	0.91 (0.81-1.03)
Oral cavity	0.91 (0.75-1.09)	0.90 (0.74-1.08)	0.93 (0.77-1.12)	0.90 (0.75-1.08)	0.90 (0.75-1.08)	0.91 (0.76-1.08)	0.94 (0.78-1.15)
Oropharynx	0.88 (0.70-1.11)	0.87 (0.69-1.10)	0.87 (0.69-1.10)	0.90 (0.71-1.13)	0.89 (0.71-1.12)	0.90 (0.72-1.13)	0.92 (0.71-1.19)
**Gastrointestinal**	**1.03 (1.00-1.07)‡**	**1.03 (1.00-1.07)‡**	1.03 (0.99-1.07)	**1.03 (1.00-1.07)‡**	1.02 (0.99-1.06)	**1.03 (1.00-1.06)‡**	1.03 (0.99-1.07)
**Oesophagus**	1.10 (0.98-1.23)	1.09 (0.97-1.22)	1.05 (0.94-1.18)	1.08 (0.97-1.21)	1.08 (0.97-1.20)	1.08 (0.97-1.21)	1.02 (0.90-1.15)
Adenocarcinoma	1.05 (0.91-1.21)	1.04 (0.89-1.20)	1.00 (0.86-1.15)	1.05 (0.91-1.20)	1.03 (0.90-1.18)	1.05 (0.91-1.20)	1.01 (0.86-1.18)
Squamous cell carcinoma	1.03 (0.79-1.34)	0.97 (0.73-1.29)	0.95 (0.72-1.25)	0.93 (0.71-1.22)	0.96 (0.74-1.25)	0.95 (0.73-1.25)	0.81 (0.60-1.11)
**Stomach**	1.08 (0.94-1.23)	1.10 (0.96-1.26)	1.11 (0.97-1.27)	1.08 (0.95-1.23)	1.08 (0.95-1.23)	1.08 (0.95-1.22)	1.05 (0.91-1.21)
Stomach cardia	0.98 (0.78-1.23)	1.01 (0.80-1.27)	1.03 (0.82-1.30)	0.99 (0.79-1.24)	0.99 (0.79-1.24)	0.98 (0.79-1.23)	1.05 (0.83-1.34)
Stomach non-cardia	1.14 (0.86-1.51)	1.12 (0.83-1.50)	1.17 (0.88-1.55)	1.19 (0.90-1.56)	1.17 (0.89-1.54)	1.17 (0.89-1.54)	1.08 (0.80-1.47)
**Small intestine**	1.22 (0.99-1.51)	1.20 (0.96-1.50)	1.16 (0.94-1.45)	1.21 (0.98-1.49)	1.21 (0.98-1.49)	1.22 (0.99-1.50)	1.08 (0.84-1.38)
**Colorectal**	1.01 (0.96-1.05)	1.01 (0.96-1.06)	1.02 (0.97-1.07)	1.02 (0.98-1.07)	1.01 (0.97-1.06)	1.02 (0.97-1.06)	1.03 (0.98-1.08)
Colon	1.03 (0.97-1.09)	1.04 (0.98-1.10)	1.05 (0.99-1.12)	1.05 (0.99-1.11)	1.04 (0.98-1.10)	1.04 (0.99-1.10)	1.05 (0.99-1.12)
Rectum	0.95 (0.88-1.04)	0.94 (0.87-1.03)	0.95 (0.87-1.03)	0.97 (0.90-1.05)	0.96 (0.89-1.04)	0.97 (0.89-1.04)	0.99 (0.91-1.08)
**Anal**	0.94 (0.73-1.21)	0.97 (0.75-1.25)	1.00 (0.77-1.29)	1.00 (0.78-1.27)	0.96 (0.75-1.24)	0.99 (0.77-1.27)	1.02 (0.78-1.35)
**Hepatobiliary tract**	1.05 (0.93-1.18)	1.06 (0.94-1.20)	1.05 (0.92-1.18)	1.02 (0.90-1.15)	1.02 (0.90-1.15)	1.03 (0.92-1.16)	1.04 (0.92-1.19)
**Liver**	1.05 (0.90-1.22)	1.08 (0.93-1.26)	1.04 (0.89-1.21)	1.03 (0.88-1.19)	1.01 (0.87-1.18)	1.04 (0.90-1.20)	1.04 (0.89-1.23)
Hepatocellular carcinoma	1.10 (0.88-1.37)	1.12 (0.89-1.40)	1.07 (0.84-1.35)	1.09 (0.87-1.36)	1.09 (0.88-1.36)	1.11 (0.90-1.37)	1.10 (0.88-1.37)
Intrahepatic bile duct	1.03 (0.80-1.33)	1.08 (0.83-1.40)	1.02 (0.79-1.33)	0.99 (0.77-1.28)	0.98 (0.76-1.26)	0.99 (0.77-1.28)	0.97 (0.73-1.28)
**Pancreas**	1.03 (0.93-1.13)	1.02 (0.92-1.13)	0.99 (0.90-1.10)	1.00 (0.91-1.10)	1.00 (0.90-1.10)	1.00 (0.91-1.10)	0.99 (0.90-1.10)
**Lung**	1.06 (0.99-1.12)	1.04 (0.98-1.11)	1.05 (0.98-1.11)	1.05 (0.99-1.11)	1.04 (0.98-1.11)	1.05 (0.99-1.11)	**1.09 (1.03-1.16)§**
**Melanoma skin**	1.01 (0.95-1.07)	1.00 (0.94-1.06)	1.00 (0.94-1.06)	1.00 (0.94-1.06)	1.00 (0.94-1.06)	1.00 (0.94-1.06)	1.00 (0.93-1.07)
**Kidney**	1.05 (0.97-1.14)	1.04 (0.95-1.13)	1.02 (0.94-1.11)	1.06 (0.98-1.15)	1.04 (0.96-1.12)	1.05 (0.97-1.13)	1.08 (0.99-1.18)
Kidney, except renal pelvis	1.06 (0.97-1.16)	1.04 (0.95-1.14)	1.04 (0.95-1.14)	1.07 (0.98-1.17)	1.05 (0.96-1.14)	1.06 (0.97-1.15)	**1.11 (1.01-1.22)‡**
Renal cell carcinoma	1.07 (0.93-1.24)	1.03 (0.89-1.20)	1.09 (0.94-1.26)	1.09 (0.94-1.25)	1.06 (0.92-1.22)	1.09 (0.95-1.25)	1.14 (0.97-1.35)
**Bladder**	1.03 (0.93-1.15)	1.02 (0.91-1.14)	1.02 (1.02-1.14)	1.00 (0.90-1.12)	1.01 (0.91-1.12)	1.01 (0.91-1.13)	1.02 (0.90-1.15)
**Brain and central nervous system**	1.08 (0.96-1.20)	1.07 (0.96-1.20)	1.06 (0.95-1.19)	1.10 (0.98-1.22)	1.08 (0.97-1.20)	1.08 (0.97-1.20)	1.08 (0.96-1.22)
Brain	1.08 (0.97-1.21)	1.08 (0.96-1.21)	1.07 (0.95-1.20)	1.10 (0.99-1.23)	1.09 (0.98-1.21)	1.08 (0.97-1.21)	1.09 (0.97-1.23)
Glioma	1.05 (0.94-1.19)	1.05 (0.93-1.19)	1.05 (0.93-1.18)	1.08 (0.96-1.21)	1.06 (0.94-1.19)	1.06 (0.94-1.18)	1.06 (0.94-1.21)
**Thyroid**	1.15 (0.99-1.35)	1.12 (0.95-1.32)	1.05 (0.89-1.23)	1.11 (0.95-1.29)	1.12 (0.96-1.30)	1.11 (0.95-1.29)	**1.20 (1.02-1.41)‡**
**Lymphatic & haematopoietic tissue**	1.01 (0.96-1.06)	1.00 (0.95-1.05)	1.00 (0.95-1.06)	1.00 (0.95-1.05)	1.00 (0.96-1.05)	1.00 (0.96-1.05)	1.00 (0.95-1.06)
**Non-Hodgkin lymphoma**	1.00 (0.94-1.06)	0.99 (0.93-1.05)	0.99 (0.94-1.05)	0.99 (0.94-1.05)	0.99 (0.94-1.05)	0.99 (0.94-1.05)	0.98 (0.92-1.04)
Diffuse large B-cell lymphoma	1.03 (0.91-1.16)	1.04 (0.91-1.18)	1.03 (0.91-1.16)	1.04 (0.92-1.17)	1.04 (0.92-1.17)	1.04 (0.92-1.17)	1.03 (0.91-1.18)
Follicular lymphoma	0.88 (0.74-1.05)	0.87 (0.73-1.04)	0.90 (0.75-1.07)	0.87 (0.74-1.04)	0.88 (0.74-1.04)	0.90 (0.76-1.06)	0.85 (0.70-1.03)
CLL/SLL	1.04 (0.92-1.17)	1.03 (0.90-1.17)	1.02 (0.90-1.16)	1.03 (0.91-1.16)	1.03 (0.91-1.16)	1.02 (0.91-1.16)	1.00 (0.87-1.15)
**Multiple myeloma**	1.01 (0.90-1.13)	0.96 (0.85-1.08)	0.97 (0.87-1.09)	0.99 (0.88-1.10)	0.99 (0.88-1.10)	0.98 (0.88-1.10)	0.99 (0.88-1.12)
**Leukaemia**	1.01 (0.92-1.11)	1.03 (0.93-1.14)	1.02 (0.92-1.12)	1.01 (0.92-1.11)	1.01 (0.92-1.11)	1.01 (0.92-1.11)	1.02 (0.92-1.13)
**Breast†**	1.00 (0.96-1.03)	1.00 (0.97-1.04)	0.99 (0.96-1.03)	1.00 (0.96-1.03)	1.00 (0.96-1.03)	1.00 (0.97-1.03)	1.00 (0.96-1.04)
Pre-menopausal breast†	1.00 (0.94-1.07)	1.01 (0.95-1.09)	1.01 (0.95-1.08)	1.00 (0.94-1.07)	1.00 (0.94-1.07)	1.01 (0.94-1.07)	1.00 (0.93-1.08)
Post-menopausal breast†	1.00 (0.95-1.05)	0.99 (0.94-1.04)	1.00 (0.95-1.04)	1.00 (0.95-1.04)	1.00 (0.95-1.04)	1.00 (0.95-1.04)	1.00 (0.95-1.05)
**Uterus†**	1.01 (0.93-1.11)	1.03 (0.94-1.12)	1.02 (0.94-1.12)	1.02 (0.94-1.11)	1.02 (0.94-1.11)	1.02 (0.94-1.11)	1.06 (0.96-1.16)
Endometrium†	1.02 (0.93-1.11)	1.03 (0.94-1.13)	1.02 (0.94-1.12)	1.03 (0.94-1.12)	1.03 (0.95-1.12)	1.03 (0.94-1.12)	1.06 (0.96-1.16)
**Ovary†**	**1.15 (1.03-1.27)§**	**1.12 (1.01-1.25)‡**	**1.16 (1.05-1.29)§**	**1.16 (1.05-1.28)§**	**1.16 (1.05-1.28)§**	**1.16 (1.05-1.28)§**	**1.17 (1.05-1.31)§**
**Prostate†**	0.99 (0.95-1.02)	0.99 (0.95-1.02)	0.99 (0.96-1.02)	0.99 (0.95-1.02)	0.99 (0.96-1.02)	0.99 (0.96-1.02)	0.99 (0.96-1.03)

Abbreviations: UPF, ultra-processed food; HR, hazard ratio; CI, confidence interval; CLL, Chronic lymphocytic leukemia; SLL, Small lymphocytic lymphoma.
All models were based on the final model and fully adjusted with age (underlying timescale), ethnicity, smoking status, physical activity level, average household income, highest educational attainment, alcohol intake, body mass index, total daily energy intake, and stratified by sex, height, family history of cancer, index of multiple deprivation quintile, and geographical region. Analyses of female-specific cancers were additionally adjusted for baseline menopausal status, use of oral contraceptives, use of hormone replacement therapy, and parity.

UPF consumption was defined as the percentage of its weight contribution relative to total food intake measured in g/day. Model results are interpreted as hazard ratio for every 10 percentage points increment in the UPF content of total diet.
Model S1 had total energy intake removed from the final model but additionally adjusted for sodium, total fat, and carbohydrate intake. Colorectal cancer outcomes were additionally adjusted for red meat, processed meat, fibre, and calcium intake.
Model S2 had total energy intake removed from the final model but additionally adjusted for sodium, trans fat, and free sugars intake. Colorectal cancer outcomes were additionally adjusted for red meat, processed meat, fibre, and calcium intake. Results were consistent when intake of saturated fat was adjusted for instead of trans fat. Intake of trans fat and saturated fat were not included in the model simultaneously due to high correlation coefficient (0.79).
Model S3 was additionally adjusted for fruit and vegetable intake.
Model S4 had alcohol intake removed from the derivation of UPF consumption and total energy intake.
Model S5 was additionally adjusted for baseline presence of diabetes, cardiovascular disease, depression, and high blood pressure.
Model S6 was additionally adjusted for number of 24-hour recalls.
Model S7 excluded participants with follow-up time <2 years (n=193878).
†Modelling for breast, uterus and ovarian cancers were conducted in women only (n=107919), modelling for prostate cancer were conducted in men only (n= 89507).
‡$P < 0.05$
§$P < 0.01$

Table S4: Results of stratified analysis for head and neck and lung cancer outcomes

Head and Neck cancer†	case/N	per 10% increment in UPF intake[b] HR (95% CI)	Quartile of UPF consumption* Q1 (lowest) Ref	Q2 HR (95% CI)	Q3 HR (95% CI)	Q4 (highest) HR (95% CI)	P_{Trend}
**Incidence by smoking status**							
Never smoked	158/111814	0·84 (0·69-1·02)	1	1·03 (0·60-1·77)	0·65 (0·35-1·19)	0·57 (0·29-1·13)	0·05
Ex-smoker	482/69545	0·87 (0·72-1·04)	1	0·61 (0·34-1·07)	0·61 (0·34-1·08)	0·60 (0·32-1·12)	0·12
Current smoker	292/15622	1·05 (0·76-1·47)	1	1·51 (0·38-5·96)	1·01 (0·24-4·19)	1·06 (0·27-4·05)	0·98
**Incidence by alcohol consumption‡**							
Non-consumer of alcohol	55/36784	0·82 (0·58-1·15)	1	0·40 (0·10-1·49)	0·51 (0·14-1·77)	0·64 (0·18-2·24)	0·63
Low alcohol consumer	113/78890	0·90 (0·75-1·08)	1	0·83 (0·43-1·60)	0·51 (0·26-1·01)	0·49 (0·23-1·00)	0·02
High alcohol consumer	174/81752	0·95 (0·79-1·14)	1	0·64 (0·40-1·04)	0·61 (0·36-1·04)	0·74 (0·41-1·34)	0·18
**Lung cancer**	**case/N**	**per 10% increment in UPF intake[b] HR (95% CI)**	**Quartile of UPF consumption* Q1 (lowest) Ref**	**Q2 HR (95% CI)**	**Q3 HR (95% CI)**	**Q4 (highest) HR (95% CI)**	**P_{Trend}**
**Incidence by smoking status**							
Never smoked	158/111814	0·94 (0·80-1·11)	1	1·02 (0·61-1·69)	0·94 (0·56-1·59)	0·92 (0·51-1·63)	0·74
Ex-smoker	482/69545	1·07 (0·97-1·17)	1	0·97 (0·71-1·31)	0·83 (0·60-1·15)	1·30 (0·94-1·80)	0·22
Current smoker	292/15622	1·05 (0·90-1·22)	1	1·26 (0·69-2·31)	1·24 (0·66-2·30)	1·26 (0·68-2·32)	0·50
**Mortality by smoking status**							
Never smoked	88/111814	1·00 (0·79-1·27)	1	1·48 (0·75-2·91)	0·85 (0·40-1·80)	1·19 (0·54-2·60)	0·95
Ex-smoker	323/69545	1·10 (0·98-1·23)	1	1·01 (0·69-1·47)	1·01 (0·68-1·50)	1·43 (0·95-2·14)	0·09
Current smoker	220/15622	1·10 (0·93-1·30)	1	1·09 (0·55-2·14)	1·23 (0·60-2·49)	1·25 (0·63-2·48)	0·48

Abbreviations: UPF, ultra-processed food; HR, hazard ratio; CI, confidence interval; Ref, reference category.

All models were fully adjusted with age (underlying timescale), ethnicity, smoking status (removed from analyses stratified by the same variable), physical activity level, average household income, highest educational attainment, alcohol intake, body mass index, total daily energy intake, and stratified by sex, height, family history of cancer, index of multiple deprivation quintile, and geographical region.

*UPF consumption was defined as the percentage of its weight contribution relative to total food intake measured in g/day. Study participants were further categorized into quartiles (Q1-Q4 represents lowest to highest quartile of UPF consumption).

†Stratified analysis for head and neck cancer mortality was not performed due to small case number in subgroups (n<20).

‡Alcohol consumption groups were defined as: non-consumer (0g/day); low alcohol consumer (<17g/day); high alcohol consumer (≥17g/day). The median alcohol consumption was 17g/day among those consumed alcohol in the study cohort.

Figure S3: Covariate adjustments for the association between UPF consumption (per 10 percentage points) and cancer-related mortality

Cancer site	Number of deaths	Model 1 Hazard Ratio (95% CI)	Model 2 Hazard Ratio (95% CI)	Model 3 Hazard Ratio (95% CI)	Model 4 Hazard Ratio (95% CI)	Model 4 P value
**All cancers**	4009	**1.08 (1.06, 1.11)**	**1.07 (1.04, 1.10)**	**1.06 (1.03, 1.08)**	**1.06 (1.03, 1.09)**	**<0.001**
**Head and Neck**	54	0.84 (0.66, 1.08)	0.90 (0.68, 1.18)	0.91 (0.69, 1.20)	0.95 (0.71, 1.27)	0.75
**Gastrointestinal**	1408	**1.07 (1.02, 1.11)**	**1.05 (1.01, 1.10)**	1.03 (0.98, 1.08)	1.04 (0.99, 1.09)	0.07
**Oesophagus**	194	1.06 (0.95, 1.18)	1.09 (0.97, 1.24)	1.07 (0.94, 1.21)	1.08 (0.95, 1.24)	0.20
**Stomach**	121	**1.23 (1.09, 1.39)**	**1.16 (1.00, 1.35)**	1.15 (0.99, 1.33)	1.16 (0.99, 1.35)	0.05
**Colorectal**	438	1.04 (0.96, 1.11)	1.02 (0.93, 1.10)	1.00 (0.92, 1.09)	1.03 (0.94, 1.12)	0.45
- Colon	244	1.05 (0.96, 1.16)	1.00 (0.89, 1.12)	0.98 (0.87, 1.11)	0.98 (0.87, 1.11)	0.86
- Rectum	194	1.01 (0.91, 1.13)	1.02 (0.90, 1.16)	1.01 (0.89, 1.14)	1.08 (0.95, 1.22)	0.23
**Hepatobiliary tract**	182	**1.13 (1.01, 1.26)**	1.08 (0.95, 1.23)	1.03 (0.90, 1.18)	1.04 (0.91, 1.19)	0.52
**Liver**	150	1.09 (0.96, 1.23)	1.06 (0.91, 1.23)	1.01 (0.87, 1.18)	1.03 (0.88, 1.20)	0.64
- Hepatocellular carcinoma	46	1.20 (0.98, 1.48)	1.16 (0.89, 1.51)	1.14 (0.87, 1.49)	1.12 (0.85, 1.49)	0.39
- Intrahepatic bile duct	103	1.04 (0.89, 1.21)	1.00 (0.83, 1.20)	0.96 (0.80, 1.16)	1.01 (0.84, 1.22)	0.86
**Pancreas**	371	1.01 (0.93, 1.10)	1.02 (0.92, 1.12)	1.00 (0.91, 1.10)	1.00 (0.90, 1.10)	0.99
**Lung**	633	**1.10 (1.04, 1.17)**	1.05 (0.98, 1.13)	1.05 (0.98, 1.13)	1.06 (0.99, 1.14)	0.07
**Melanoma skin**	63	1.05 (0.86, 1.27)	1.10 (0.87, 1.38)	1.11 (0.88, 1.40)	1.15 (0.89, 1.49)	0.25
**Kidney**	117	1.08 (0.94, 1.24)	1.06 (0.91, 1.24)	1.04 (0.89, 1.21)	1.04 (0.89, 1.22)	0.57
**Bladder**	112	1.08 (0.94, 1.25)	1.04 (0.87, 1.24)	1.03 (0.86, 1.23)	1.05 (0.87, 1.25)	0.59
**Brain and central nervous system**	251	1.04 (0.94, 1.14)	1.05 (0.94, 1.18)	1.05 (0.94, 1.17)	1.05 (0.93, 1.18)	0.39
**Lymphatic & haematopoietic tissue**	376	**1.12 (1.04, 1.21)**	1.08 (0.98, 1.18)	1.06 (0.97, 1.16)	1.05 (0.95, 1.15)	0.29
**Non-Hodgkin lymphoma**	141	1.10 (0.97, 1.25)	1.02 (0.87, 1.19)	1.02 (0.87, 1.19)	1.02 (0.87, 1.20)	0.75
**Multiple myeloma**	91	**1.19 (1.03, 1.38)**	1.18 (0.98, 1.41)	1.15 (0.96, 1.38)	1.08 (0.89, 1.31)	0.42
**Leukaemia**	137	1.08 (0.95, 1.23)	1.04 (0.90, 1.21)	1.02 (0.88, 1.19)	1.02 (0.87, 1.19)	0.78
**Breast**	176	1.09 (0.98, 1.21)	**1.17 (1.03, 1.32)**	**1.14 (1.01, 1.29)**	**1.16 (1.02, 1.32)**	**0.02**
- Post-menopausal	120	1.13 (0.98, 1.29)	1.14 (0.97, 1.33)	1.10 (0.94, 1.29)	1.11 (0.94, 1.31)	0.18
**Uterus**	61	1.01 (0.82, 1.23)	1.08 (0.83, 1.40)	1.06 (0.81, 1.39)	1.01 (0.76, 1.33)	0.93
- Endometrium	43	0.99 (0.77, 1.26)	1.06 (0.77, 1.46)	1.04 (0.75, 1.44)	0.99 (0.70, 1.40)	0.99
**Ovary**	143	**1.24 (1.11, 1.39)**	**1.31 (1.14, 1.50)**	**1.30 (1.13, 1.49)**	**1.30 (1.13, 1.50)**	**<0.001**
**Prostate**	194	1.02 (0.91, 1.15)	0.97 (0.85, 1.11)	0.97 (0.85, 1.10)	0.98 (0.85, 1.12)	0.79

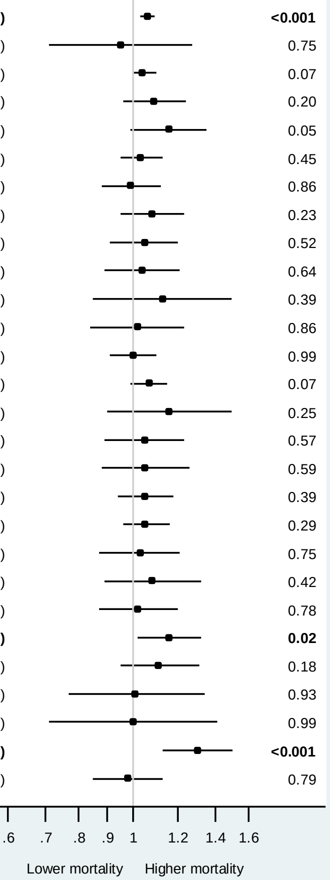

Abbreviations: UPF, ultra-processed food; CI, confidence interval.

UPF consumption was defined as the percentage of its weight contribution relative to total food intake measured in g/day. Model results are interpreted as hazard ratio for every 10 percentage points increment in the UPF content of total diet. Model 1 included age (underlying timescale), and stratified by sex. Model 2 additionally included ethnicity, smoking status, physical activity level, average household income, highest educational attainment, alcohol intake, and additionally stratified by height, family history of cancer, index of multiple deprivation quintile, and geographical region. Female-specific cancer outcomes were additionally adjusted for baseline menopausal status, use of oral contraceptives, use of hormone replacement therapy, and parity. Model 3 additionally included body mass index category. Model 4 additionally included total daily energy intake. Analysis for risk of breast, uterus and ovarian cancers were conducted in women only (n=107919) and risk of prostate cancer was conducted in men only (n= 89507).

Table S5: Sensitivity analyses for the association between UPF consumption (per 10 percentage points) and cancer-related mortality

**Cancer site**	**Model S1 HR (95% CI)**	**Model S2 HR (95% CI)**	**Model S3 HR (95% CI)**	**Model S4 HR (95% CI)**	**Model S5 HR (95% CI)**	**Model S6 HR (95% CI)**	**Model S7 HR (95% CI)**
**All cancer**	**1.05 (1.02-1.09)¶**	**1.05 (1.02-1.08)§**	**1.05 (1.02-1.08)¶**	**1.06 (1.03-1.09)¶**	**1.05 (1.02-1.08)¶**	**1.06 (1.03-1.09)¶**	**1.06 (1.03-1.09) ¶**
**Head and Neck**	0.95 (0.70-1.29)	0.94 (0.68-1.29)	0.90 (0.67-1.22)	0.94 (0.70-1.27)	0.95 (0.71-1.27)	0.97 (0.72-1.29)	0.99 (0.74-1.33)
**Gastrointestinal**	1.04 (0.99-1.10)	1.04 (0.99-1.10)	1.03 (0.98-1.09)	1.04 (0.99-1.09)	1.03 (0.98-1.08)	1.04 (0.99-1.09)	1.04 (0.99-1.09)
**Oesophagus**	1.10 (0.96-1.26)	1.08 (0.94-1.25)	1.08 (0.94-1.24)	1.08 (0.94-1.23)	1.08 (0.94-1.23)	1.08 (0.95-1.23)	1.06 (0.93-1.22)
**Stomach**	**1.18 (1.00-1.38)‡**	**1.20 (1.01-1.42)‡**	**1.17 (1.00-1.38)‡**	**1.17 (1.00-1.36)‡**	**1.18 (1.00-1.38)‡**	1.15 (0.99-1.34)	1.12 (0.95-1.33)
**Colorectal**	0.98 (0.89-1.08)	0.99 (0.90-1.09)	1.02 (0.93-1.12)	1.03 (0.94-1.13)	1.02 (0.94-1.12)	1.03 (0.94-1.12)	1.04 (0.95-1.14)
Colon	0.93 (0.82-1.06)	0.95 (0.83-1.08)	0.99 (0.88-1.13)	0.99 (0.88-1.12)	0.97 (0.86-1.10)	0.99 (0.88-1.12)	1.01 (0.89-1.15)
Rectum	1.05 (0.91-1.20)	1.06 (0.92-1.22)	1.05 (0.92-1.20)	1.07 (0.94-1.22)	1.07 (0.94-1.22)	1.07 (0.94-1.21)	1.08 (0.95-1.23)
**Hepatobiliary tract**	1.05 (0.91-1.21)	1.05 (0.91-1.22)	1.03 (0.89-1.19)	1.04 (0.90-1.19)	1.04 (0.90-1.19)	1.04 (0.91-1.19)	1.04 (0.90-1.20)
**Liver**	1.05 (0.89-1.22)	1.05 (0.89-1.24)	1.01 (0.86-1.19)	1.02 (0.87-1.20)	1.03 (0.88-1.20)	1.03 (0.88-1.20)	1.04 (0.88-1.22)
Hepatocellular carcinoma	1.12 (0.84-1.49)	1.12 (0.83-1.49)	1.06 (0.78-1.43)	1.11 (0.82-1.49)	1.12 (0.84-1.51)	1.10 (0.83-1.44)	1.15 (0.86-1.52)
Intrahepatic bile duct	1.03 (0.85-1.25)	1.05 (0.85-1.29)	1.01 (0.83-1.24)	1.01 (0.83-1.23)	1.00 (0.82-1.21)	1.02 (0.84-1.23)	1.01 (0.83-1.24)
**Pancreas**	1.02 (0.92-1.12)	1.01 (0.91-1.12)	1.00 (0.90-1.10)	0.99 (0.90-1.10)	0.99 (0.90-1.09)	1.00 (0.90-1.10)	1.00 (0.90-1.11)
**Lung**	1.05 (0.98-1.14)	1.05 (0.97-1.14)	1.06 (0.98-1.14)	1.06 (0.98-1.14)	1.06 (0.98-1.14)	1.06 (0.99-1.14)	**1.09 (1.01-1.18)‡**
**Melanoma skin**	1.09 (0.84-1.43)	1.09 (0.82-1.44)	1.08 (0.82-1.41)	1.17 (0.90-1.51)	1.16 (0.90-1.50)	1.16 (0.90-1.49)	1.06 (0.81-1.39)
**Kidney**	1.03 (0.87-1.21)	1.00 (0.83-1.19)	0.97 (0.82-1.16)	1.06 (0.90-1.25)	1.02 (0.87-1.21)	1.04 (0.88-1.22)	1.04 (0.87-1.23)
**Bladder**	1.02 (0.85-1.23)	1.03 (0.85-1.25)	1.04 (0.86-1.26)	1.03 (0.86-1.24)	1.05 (0.88-1.26)	1.04 (0.87-1.25)	1.03 (0.85-1.23)
**Brain and central nervous system**	1.03 (0.91-1.16)	1.03 (0.91-1.16)	1.05 (0.93-1.18)	1.07 (0.95-1.20)	1.05 (0.93-1.17)	1.04 (0.93-1.17)	1.04 (0.92-1.18)
**Lymphatic & haematopoietic tissue**	1.03 (0.94-1.14)	1.03 (0.93-1.15)	1.08 (0.98-1.20)	1.05 (0.95-1.16)	1.05 (0.95-1.15)	1.04 (0.95-1.15)	1.05 (0.95-1.15)
**Non-Hodgkin lymphoma**	0.99 (0.84-1.17)	0.99 (0.83-1.19)	1.06 (0.89-1.26)	1.03 (0.87-1.21)	1.01 (0.86-1.19)	1.02 (0.86-1.19)	1.01 (0.85-1.19)
**Multiple myeloma**	1.09 (0.89-1.33)	1.04 (0.84-1.29)	1.11 (0.90-1.37)	1.08 (0.88-1.32)	1.12 (0.92-1.38)	1.06 (0.87-1.30)	1.09 (0.90-1.33)
**Leukaemia**	1.00 (0.85-1.18)	1.02 (0.86-1.21)	1.05 (0.89-1.24)	1.02 (0.86-1.19)	1.02 (0.87-1.19)	1.02 (0.87-1.19)	1.03 (0.87-1.21)
**Breast**	**1.15 (1.00-1.31)‡**	**1.15 (1.01-1.33)‡**	**1.13 (1.00-1.30)‡**	**1.16 (1.02-1.32)‡**	**1.16 (1.02-1.32)‡**	**1.16 (1.02-1.32)‡**	**1.19 (1.05-1.36)§**
Post-menopausal	1.10 (0.93-1.31)	1.12 (0.93-1.35)	1.11 (0.93-1.32)	1.11 (0.94-1.32)	1.11 (0.95-1.31)	1.14 (0.96-1.35)	1.13 (0.96-1.34)
**Uterus**	1.01 (0.76-1.35)	1.00 (0.74-1.35)	0.97 (0.72-1.31)	1.01 (0.76-1.33)	1.01 (0.76-1.33)	0.99 (0.74-1.32)	1.01 (0.76-1.33)
Endometrium	0.96 (0.66-1.38)	0.94 (0.64-1.38)	0.89 (0.60-1.31)	1.00 (0.70-1.41)	0.97 (0.69-1.38)	0.97 (0.68-1.39)	0.98 (0.69-1.39)
**Ovary**	**1.25 (1.08-1.45)§**	**1.26 (1.08-1.48)§**	**1.29 (1.11-1.50)§**	**1.30 (1.13-1.51)¶**	**1.31 (1.13-1.51)¶**	**1.29 (1.11-1.48)¶**	**1.28 (1.10-1.48)§**
**Prostate**	0.97 (0.85-1.12)	0.95 (0.82-1.11)	0.99 (0.86-1.15)	0.98 (0.85-1.13)	0.97 (0.85-1.12)	0.98 (0.85-1.12)	0.96 (0.83-1.11)

Abbreviations: UPF, ultra-processed food; HR, hazard ratio; CI, confidence interval.

All models were based on the final model and fully adjusted with age (underlying timescale), ethnicity, smoking status, physical activity level, average household income, highest educational attainment, alcohol intake, body mass index, total daily energy intake, and stratified by sex, height, family history of cancer, index of multiple deprivation quintile, and geographical region. Analyses of female-specific cancers were additionally adjusted for baseline menopausal status, use of oral contraceptives, use of hormone replacement therapy, and parity.

UPF consumption was defined as the percentage of its weight contribution relative to total food intake measured in g/day. Model results are interpreted as hazard ratio for every 10 percentage points increment in UPF content of total diet.

Model S1 had total energy intake removed from the final model but additionally adjusted for sodium, total fat, and carbohydrate intake. Colorectal cancer outcomes were additionally adjusted for red meat, processed meat, fibre, and calcium intake.

Model S2 had total energy intake removed from the final model but additionally adjusted for sodium, trans fat, and free sugars intake. Colorectal cancer outcomes were additionally adjusted for red meat, processed meat, fibre, and calcium intake. Results were consistent when intake of saturated fat was adjusted for instead of trans fat. Intake of trans fat and saturated fat were not included in the model simultaneously due to high correlation coefficient (0.79).

Model S3 was additionally adjusted for fruit and vegetable intake.

Model S4 had alcohol intake removed from the derivation of UPF consumption and total energy intake.

Model S5 was additionally adjusted for baseline presence of diabetes, cardiovascular disease, depression, and high blood pressure.

Model S6 was additionally adjusted for number of 24-hour recalls.

Model S7 excluded participants with follow-up time <2 years (n=196657).

†Modelling for breast, uterus and ovarian cancers were conducted in women only (n=107919), modelling for prostate cancer were conducted in men only (n= 89507).
‡$P < 0.05$
§$P < 0.01$
¶$P < 0.001$

Figure S4: Sources of NOVA subgroups based on total energy intake by quartile of UPF consumption

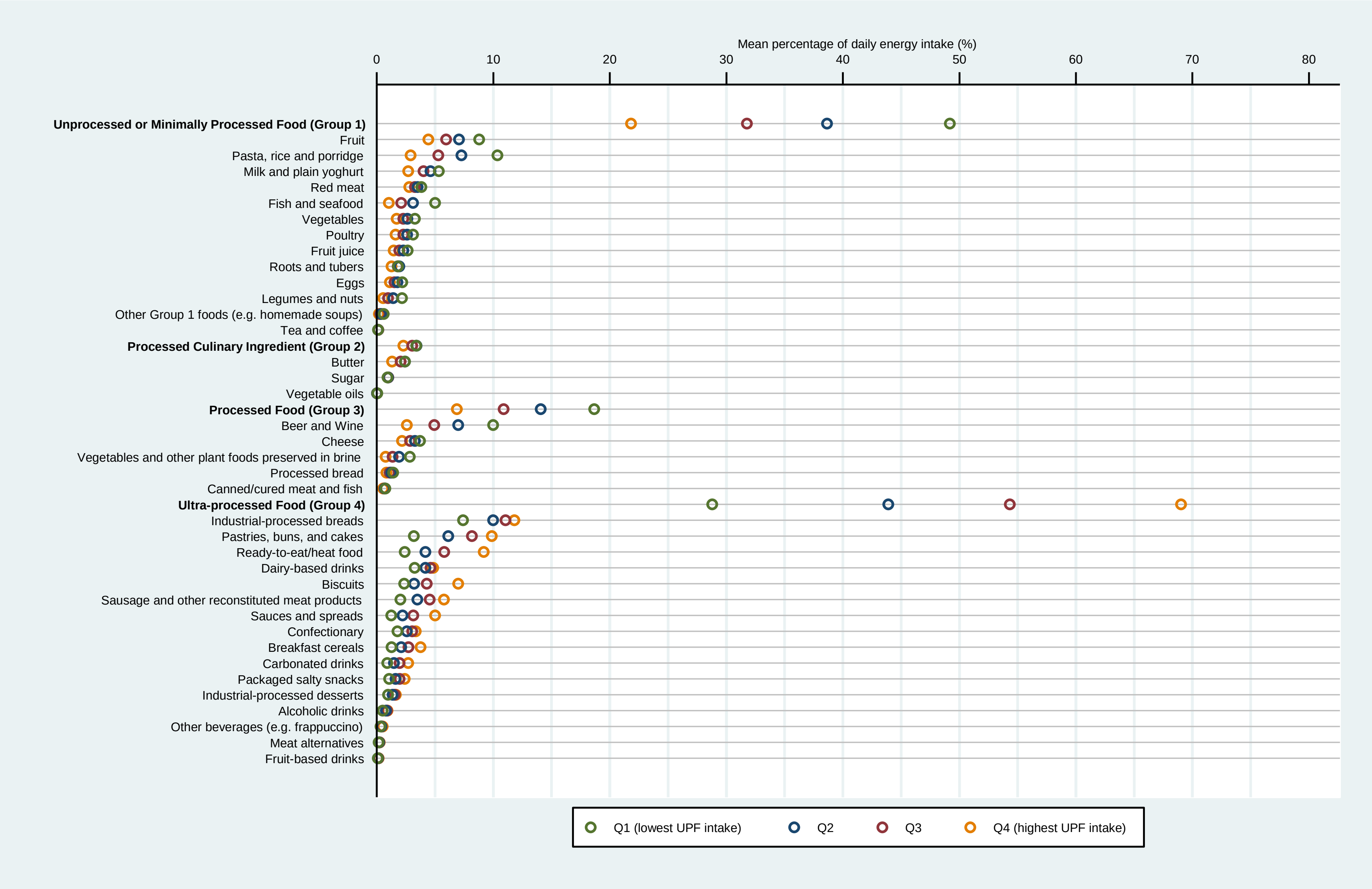

Abbreviations: UPF, ultra-processed food.

UPF consumption was defined as the percentage of its calorie contribution relative to total energy intake measured in kcal/day. Study participants were further categorized into quartiles (Q1-Q4 represents lowest to highest quartile of UPF consumption).

Table S6: Association of cancer incidence by levels of UPF consumption based on total energy intake

Cancer site	Number of incident cases	per 10% increment in UPF intake* HR (95% CI)	Quartile of UPF consumption* Q1 (lowest) Ref	Q2 HR (95% CI)	Q3 HR (95% CI)	Q4 (highest) HR (95% CI)	P_{Trend}
**All cancers**	15921	**1·01 (1·00-1·02)‡**	1	1·04 (0·99-1·09)	1·02 (0·97-1·07)	**1·05 (1·00-1·11)‡**	0·08
**Head and Neck**	342	0·91 (0·85-0·99)	1	**0·67 (0·49-0·93)‡**	**0·58 (0·41-0·82)§**	0·77 (0·55-1·07)	0·08
Oral cavity	106	0·94 (0·82-1·08)	1	0·90 (0·49-1·62)	1·00 (0·55-1·84)	0·95 (0·51-1·75)	0·95
Oropharynx	86	0·92 (0·78-1·08)	1	0·72 (0·38-1·35)	**0·43 (0·21-0·90)‡**	0·69 (0·35-1·36)	0·14
**Gastrointestinal**	2937	1·01 (0·98-1·03)	1	0·97 (0·87-1·09)	1·03 (0·91-1·15)	0·98 (0·87-1·11)	0·91
**Oesophagus**	283	1·07 (0·97-1·17)	1	1·21 (0·84-1·76)	1·18 (0·81-1·73)	1·24 (0·83-1·85)	0·33
Adenocarcinoma	186	1·02 (0·91-1·14)	1	1·19 (0·75-1·88)	1·21 (0·75-1·93)	1·03 (0·63-1·70)	0·87
Squamous cell carcinoma	67	1·07 (0·89-1·30)	1	1·55 (0·73-3·29)	0·99 (0·43-2·27)	1·54 (0·67-3·52)	0·53
**Stomach**	189	1·03 (0·93-1·15)	1	1·04 (0·65-1·68)	1·45 (0·92-2·30)	1·00 (0·61-1·64)	0·68
Stomach cardia	75	1·02 (0·85-1·21)	1	0·96 (0·45-2·04)	1·36 (0·67-2·73)	0·80 (0·35-1·79)	0·90
Stomach non-cardia	48	1·01 (0·81-1·25)	1	0·99 (0·39-2·52)	1·10 (0·43-2·80)	0·88 (0·33-2·33)	0·85
**Small intestine**	77	1·16 (0·98-1·38)	1	**2·35 (1·04-5·31)‡**	**2·63 (1·18-5·85)‡**	2·13 (0·90-5·02)	0·09
**Colorectal**	1670	1·00 (0·97-1·04)	1	0·91 (0·78-1·05)	0·97 (0·84-1·13)	0·99 (0·84-1·16)	0·88
Colon	1091	1·02 (0·98-1·07)	1	0·90 (0·74-1·09)	1·01 (0·84-1·22)	1·06 (0·87-1·29)	0·35
Rectum	579	0·96 (0·91-1·03)	1	0·92 (0·72-1·18)	0·90 (0·70-1·17)	0·85 (0·65-1·12)	0·28
**Anal**	60	1·10 (0·91-1·33)	1	1·65 (0·73-3·70)	1·45 (0·61-3·43)	1·94 (0·83-4·57)	0·18
**Hepatobiliary tract**	243	0·99 (0·89-1·09)	1	1·06 (0·70-1·59)	1·05 (0·69-1·59)	0·91 (0·58-1·42)	0·69
**Liver**	157	0·99 (0·88-1·13)	1	0·80 (0·48-1·35)	1·00 (0·60-1·66)	0·84 (0·49-1·46)	0·76
Hepatocellular carcinoma	74	1·09 (0·90-1·31)	1	1·58 (0·67-3·70)	1·79 (0·78-4·12)	1·53 (0·64-3·65)	0·37
Intrahepatic bile duct	67	0·88 (0·71-1·08)	1	0·50 (0·23-1·09)	0·50 (0·22-1·12)	0·50 (0·20-1·20)	0·11
**Pancreas**	386	0·95 (0·88-1·03)	1	0·86 (0·63-1·17)	0·82 (0·60-1·14)	0·73 (0·52-1·02)	0·07
**Lung**	935	1·02 (0·97-1·07)	1	0·94 (0·75-1·16)	1·09 (0·88-1·34)	1·14 (0·92-1·42)	0·12
**Melanoma skin**	974	1·01 (0·97-1·06)	1	1·07 (0·88-1·30)	0·94 (0·77-1·15)	**1·26 (1·02-1·54)‡**	0·09
**Kidney**	451	1·00 (0·93-1·07)	1	1·02 (0·76-1·37)	0·93 (0·69-1·26)	0·97 (0·71-1·32)	0·74
Kidney, except renal pelvis	413	1·00 (0·93-1·07)	1	1·01 (0·74-1·38)	0·97 (0·71-1·32)	0·96 (0·69-1·32)	0·74
Renal cell carcinoma	130	1·09 (0·96-1·24)	1	1·36 (0·74-2·52)	1·55 (0·86-2·80)	1·56 (0·85-2·83)	0·15
**Bladder**	320	1·00 (0·91-1·09)	1	1·11 (0·77-1·59)	1·29 (0·89-1·85)	0·93 (0·63-1·39)	0·95
**Brain and central nervous system**	284	1·03 (0·94-1·13)	1	1·06 (0·74-1·53)	1·01 (0·70-1·47)	1·28 (0·87-1·87)	0·26
Brain	277	1·03 (0·94-1·13)	1	1·05 (0·72-1·51)	1·03 (0·71-1·50)	1·26 (0·85-1·85)	0·28
Glioma	242	1·02 (0·93-1·13)	1	1·19 (0·80-1·76)	1·07 (0·71-1·61)	1·21 (0·79-1·84)	0·49
**Thyroid**	126	1.06 (0·93-1·22)	1	1·41 (0·78-2·53)	1·69 (0·94-3·04)	1·25 (0·66-2·35)	0·42
**Lymphatic & haematopoietic tissue**	1429	**1·04 (1·00-1·08)‡**	1	1·13 (0·96-1·33)	1·05 (0·89-1·24)	**1·19 (1·00-1·41)‡**	0·11
**Non-Hodgkin lymphoma**	1091	1·03 (0·98-1·07)	1	1·12 (0·93-1·35)	1·01 (0·83-1·22)	1·13 (0·93-1·38)	0·39
Diffuse large B-cell lymphoma	210	1·05 (0·95-1·16)	1	**1·73 (1·11-2·69)‡**	1·25 (0·77-2·01)	1·43 (0·88-2·31)	0·47
Follicular lymphoma	154	1·00 (0·88-1·13)	1	1·03 (0·63-1·69)	0·91 (0·54-1·54)	1·17 (0·68-2·00)	0·68
CLL/SLL	226	1·01 (0·92-1·11)	1	0·79 (0·53-1·20)	1·01 (0·68-1·50)	1·00 (0·66-1·51)	0·74
**Multiple myeloma**	286	1·04 (0·95-1·13)	1	1·22 (0·84-1·78)	0·93 (0·63-1·38)	1·24 (0·84-1·83)	0·56
**Leukaemia**	400	1·01 (0·94-1·09)	1	0·80 (0·58-1·09)	1·08 (0·80-1·46)	0·98 (0·71-1·35)	0·60
**Breast†**	3030	1·00 (0·97-1·02)	1	1·07 (0·96-1·19)	1·05 (0·94-1·18)	1·02 (0·91-1·15)	0·68
Pre-menopausal breast†	717	0·98 (0·93-1·04)	1	1·18 (0·93-1·50)	0·97 (0·76-1·25)	1·03 (0·80-1·32)	0·79
Post-menopausal breast†	1856	1·01 (0·97-1·04)	1	1·04 (0·91-1·19)	1·06 (0·92-1·23)	1·03 (0·89-1·20)	0·55
**Uterus†**	439	1·02 (0·96-1·10)	1	1·09 (0·82-1·46)	1·05 (0·78-1·41)	1·25 (0·93-1·69)	0·19
Endometrium†	429	1·03 (0·96-1·11)	1	1·13 (0·84-1·52)	1·07 (0·79-1·44)	1·28 (0·94-1·74)	0·16
**Ovary†**	291	**1·11 (1·02-1·22)‡**	1	1·35 (0·92-1·98)	1·45 (0·99-2·12)	1·41 (0·94-2·10)	0·09
**Prostate†**	3621	0·99 (0·97-1·02)	1	1·02 (0·92-1·13)	0·94 (0·85-1·04)	0·98 (0·88-1·09)	0·44

Abbreviations: UPF, ultra-processed food; HR, hazard ratio; CI, confidence interval; Ref, reference category; CLL, Chronic lymphocytic leukemia; SLL, Small lymphocytic lymphoma.

All models were fully adjusted with age (underlying timescale), ethnicity, smoking status, physical activity level, average household income, highest educational attainment, alcohol intake, body mass index, and stratified by sex, height, family history of cancer, index of multiple deprivation quintile, and geographical region. Analyses of female-specific cancers were additionally adjusted for baseline menopausal status, use of oral contraceptives, use of hormone replacement therapy, and parity.

[*]UPF consumption was defined as the percentage of its calorie contribution relative to total energy intake measured in kcal/day. Study participants were further categorized into quartiles (Q1-Q4 represents lowest to highest quartile of UPF consumption).

†Modelling for breast, uterus and ovarian cancers were conducted in women only (n=107919), modelling for prostate cancer were conducted in men only (n= 89507).

‡$P < 0.05$

§$P < 0.01$

Table S7: Association of cancer-related mortality by levels of UPF consumption based on total energy intake

Cancer site	Number of deaths	per 10% increment in UPF intake* HR (95% CI)	Quartile of UPF consumption* Q1 (lowest) Ref	Q2 HR (95% CI)	Q3 HR (95% CI)	Q4 (highest) HR (95% CI)	P_{Trend}
**All cancers**	4009	**1·03 (1·00-1·05)**§	1	**1·10 (1·00-1·22)**‡	1·10 (0·99-1·21)	**1·13 (1·01-1·25)**‡	**0·03**
**Head and Neck**	54	1·01 (0·81-1·26)	1	0·40 (0·14-1·13)	1·12 (0·47-2·67)	1·24 (0·50-3·05)	0·33
**Gastrointestinal**	1408	1·00 (0·96-1·04)	1	1·05 (0·89-1·24)	1·01 (0·86-1·20)	0·96 (0·80-1·15)	0·62
**Oesophagus**	194	**1·14 (1·02-1·28)**‡	1	**1·68 (1·03-2·71)**‡	**1·70 (1·04-2·79)**‡	**1·74 (1·04-2·91)**‡	0·05
**Stomach**	121	1·01 (0·89-1·16)	1	1·39 (0·77-2·51)	1·14 (0·62-2·10)	1·11 (0·60-2·07)	0·96
**Colorectal**	438	0·98 (0·91-1·05)	1	1·01 (0·75-1·35)	0·98 (0·73-1·32)	0·88 (0·64-1·22)	0·47
Colon	244	1·02 (0·92-1·12)	1	0·97 (0·64-1·47)	1·22 (0·82-1·82)	0·91 (0·58-1·42)	0·99
Rectum	194	0·94 (0·84-1·04)	1	1·05 (0·69-1·60)	0·69 (0·43-1·10)	0·85 (0·53-1·35)	0·24
**Hepatobiliary tract**	182	1·00 (0·89-1·13)	1	0·87 (0·53-1·43)	1·04 (0·64-1·70)	0·87 (0·52-1·47)	0·83
**Liver**	150	0·97 (0·86-1·11)	1	0·76 (0·45-1·30)	0·85 (0·50-1·45)	0·73 (0·41-1·29)	0·39
Hepatocellular carcinoma	46	1·10 (0·85-1·41)	1	1·22 (0·40-3·66)	1·54 (0·50-4·76)	1·31 (0·41-4·20)	0·59
Intrahepatic bile duct	103	0·93 (0·79-1·08)	1	0·61 (0·32-1·15)	0·63 (0·33-1·18)	0·58 (0·29-1·16)	0·14
**Pancreas**	371	0·95 (0·88-1·02)	1	0·87 (0·64-1·19)	0·78 (0·56-1·08)	0·73 (0·52-1·03)	0·05
**Lung**	633	1·05 (0·99-1·12)	1	1·02 (0·78-1·33)	1·18 (0·90-1·53)	1·27 (0·97-1·67)	0·05
**Melanoma skin**	63	1·11 (0·91-1·34)	1	1·34 (0·61-2·92)	0·60 (0·24-1·52)	1·72 (0·75-3·97)	0·54
**Kidney**	117	1·03 (0·90-1·18)	1	1·11 (0·63-1·96)	1·06 (0·58-1·90)	1·18 (0·65-2·15)	0·64
**Bladder**	112	1·01 (0·86-1·18)	1	0·73 (0·40-1·33)	0·80 (0·43-1·49)	0·85 (0·45-1·62)	0·76
**Brain and central nervous system**	251	1·02 (0·93-1·12)	1	0·94 (0·64-1·38)	0·98 (0·66-1·44)	1·14 (0·76-1·71)	0·49
**Lymphatic & haematopoietic tissue**	376	1·07 (0·99-1·16)	1	1·24 (0·88-1·74)	1·25 (0·88-1·78)	1·38 (0·97-1·97)	0·09
**Non-Hodgkin lymphoma**	141	1·13 (0·99-1·29)	1	**1·87 (1·04-3·37)**‡	1·52 (0·81-2·83)	**2·05 (1·11-3·81)**‡	0·07
**Multiple myeloma**	91	1·14 (0·96-1·35)	1	2·12 (0·98-4·58)	1·56 (0·69-3·51)	**2·38 (1·07-5·29)**‡	0·09
**Leukaemia**	137	0·96 (0·84-1·10)	1	0·70 (0·41-1·22)	1·05 (0·62-1·78)	0·73 (0·41-1·29)	0·57
**Breast**†	176	1·02 (0·91-1·14)	1	1·02 (0·65-1·61)	1·24 (0·78-1·97)	1·17 (0·71-1·94)	0·40
Post-menopausal†	120	0·98 (0·85-1·14)	1	0·85 (0·48-1·51)	1·18 (0·66-2·13)	1·01 (0·53-1·93)	0·75
**Uterus**†	61	0·99 (0·79-1·22)	1	1·00 (0·44-2·26)	0·78 (0·32-1·89)	1·08 (0·43-2·68)	0·95
Endometrium†	43	0·99 (0·76-1·29)	1	0·98 (0·37-2·61)	0·94 (0·32-2·71)	1·07 (0·34-3·29)	0·93
**Ovary**†	143	**1·20 (1·05-1·37)**§	1	1·48 (0·84-2·62)	1·59 (0·90-2·81)	1·79 (1·00-3·23)	0·06
**Prostate**†	194	0·94 (0·84-1·06)	1	1·04 (0·67-1·63)	0·84 (0·53-1·35)	0·96 (0·59-1·56)	0·68

Abbreviations: UPF, ultra-processed food; HR, hazard ratio; CI, confidence interval; Ref, reference category; CLL, Chronic lymphocytic leukemia; SLL, Small lymphocytic lymphoma.
All models were fully adjusted with age (underlying timescale), ethnicity, smoking status, physical activity level, average household income, highest educational attainment, alcohol intake, body mass index, and stratified by sex, height, family history of cancer, index of multiple deprivation quintile, and geographical region. Analyses of female-specific cancers were additionally adjusted for baseline menopausal status, use of oral contraceptives, use of hormone replacement therapy, and parity.
*UPF consumption was defined as the percentage of its calorie contribution relative to total energy intake measured in kcal/day. Study participants were further categorized into quartiles (Q1-Q4 represents lowest to highest quartile of UPF consumption).
†Modelling for breast, uterus and ovarian cancers were conducted in women only (n=107919), modelling for prostate cancer were conducted in men only (n= 89507).
‡$P < 0.05$
§$P < 0.01$
